# Antibody drug conjugates against the receptor for advanced glycation end products (RAGE), a novel therapeutic target in endometrial cancer

**DOI:** 10.1186/s40425-019-0765-z

**Published:** 2019-10-29

**Authors:** Gareth D. Healey, Belen Pan-Castillo, Jezabel Garcia-Parra, Julia Davies, Shaun Roberts, Eilir Jones, Kalyan Dhar, Sarika Nandanan, Nasima Tofazzal, Luke Piggott, Richard Clarkson, Gillian Seaton, Asa Frostell, Tim Fagge, Colin McKee, Lavinia Margarit, R. Steven Conlan, Deyarina Gonzalez

**Affiliations:** 1 Reproductive Biology and Gynaecological Oncology Group, Swansea University Medical School, Swansea University, Singleton Park, Swansea, SA2 8PP UK; 2 Cellular Pathology Department, Swansea Bay University Health Board, Singleton Hospital, Sketty Lane, Swansea, SA2 8QA UK; 3 Gynecology Oncology Department, Swansea Bay University Health Board, Singleton Hospital, Sketty Lane, Swansea, SA2 8QA UK; 4 Obstetrics & Gynecology Department Princess of Wales Hospital, Cwm Taf Morgannwg University Health Board, Coity Road, Bridgend, CF31 1RQ UK; 5 Welsh Cancer Research Centre, Institute of Cancer & Genetics, School of Medicine, Cardiff University, University Hospital of Wales, Heath Park, Cardiff, CF14 4XN UK; 6 European Cancer Stem Cell Research Institute, School of Biosciences, Cardiff University, Hadyn Ellis Building, Maindy Road, Cathays, Cardiff, CF24 4HQ UK; 7 GE Healthcare Bio-Sciences, SE-751 84 Uppsala, Sweden; 8 GE Healthcare, Little Chalfont, Buckinghamshire HP7 9NA UK; 9 ADC Biotechnology Ltd, OpTIC Technium, Ffordd William Morgan, St Asaph Business Park, St Asaph, Denbighshire LL17 0JD UK

**Keywords:** Endometrial cancer, Therapeutic, Antibody drug conjugates, RAGE, Human

## Abstract

**Background:**

The treatment of endometrial cancer (EC), the most common gynecological cancer, is currently hampered by the toxicity of current cytotoxic agents, meaning novel therapeutic approaches are urgently required.

**Methods:**

A cohort of 161 patients was evaluated for the expression of the receptor for advanced glycation end products (RAGE) in endometrial tissues. The present study also incorporates a variety of in vitro methodologies within multiple cell lines to evaluate RAGE expression and antibody-drug conjugate efficacy, internalisation and intercellular trafficking. Additionally, we undertook in vivo bio-distribution and toxicity evaluation to determine the suitability of our chosen therapeutic approach, together with efficacy studies in a mouse xenograft model of disease.

**Results:**

We have identified an association between over-expression of the receptor for advanced glycation end products (RAGE) and EC (H-score = Healthy: 0.46, SD 0.26; Type I EC: 2.67, SD 1.39; Type II EC: 2.20, SD 1.34; ANOVA, *p* < 0.0001). Furthermore, increased expression was negatively correlated with patient survival (Spearman’s Rank Order Correlation: ρ = − 0.3914, *p* < 0.05). To exploit this association, we developed novel RAGE-targeting antibody drug conjugates (ADC) and demonstrated the efficacy of this approach. RAGE-targeting ADCs were up to 100-fold more efficacious in EC cells compared to non-malignant cells and up to 200-fold more cytotoxic than drug treatment alone. Additionally, RAGE-targeting ADCs were not toxic in an in vivo pre-clinical mouse model, and significantly reduced tumour growth in a xenograft mouse model of disease.

**Conclusions:**

These data, together with important design considerations implied by the present study, suggest RAGE-ADCs could be translated to novel therapeutics for EC patients.

## One sentence summary

The Receptor for Advanced Glycation End products is differentially expressed in endometrial cancers and druggable via an antibody drug conjugate therapeutic approach.

## Background

Gynecological cancers, encompassing cancers of the endometrium, uterus, ovaries, cervix, vulva and vagina, cause significant morbidity and mortality. By 2020, estimates suggest there will be 892,000 new cases of gynecological cancer annually, worldwide, leading to 499,000 deaths [1]. Treatment is complicated by the nonspecific and highly-toxic nature of current anti-cancer drugs, such as DNA alkylating agents or platinum-based drugs used to treat this diseases, necessitating suboptimal dosing to reduce toxicity in normal cells and risking the emergence of drug-resistance in cancer cells.

Endometrial cancer (EC), is the most frequently occurring gynecological cancer in developed countries with over 319,000 cases diagnosed worldwide, and in excess of 76,000 deaths annually [2]. Morphological classification of EC into estrogen dependent (Type I) and estrogen independent cancers (Type II) reflects fundamental differences in the causes of each sub-type [2–4]. Type I EC (80–90% of EC) is primarily due to unopposed estrogenic stimulation (obesity, Polycystic Ovary Syndrome, tamoxifen), and other risk factors such as early menarche, late menopause or nulliparity [3, 5]. Type II EC (10–20% of EC) occurs mostly in older, multiparous women of normal weight [3].

Despite increasing molecular knowledge of the tumorigenesis of EC, the primary treatment option for type I and type II EC is still surgery to remove the tumor [6]. Indications for radiotherapry are limited, and even then only considered in an adjuvant setting. In advanced stage type I disease and type II EC, adjuvant chemotherapy can be advantageous [7], but many women with advanced, metastatic EC are elderly and may have undergone radiation therapy meaning they are especially susceptible to the adverse effects of aggressive cytotoxic regimens [8]. In addition, type II EC tumors typically do not respond to hormone therapies due to a lack of ER and PR expression [9], meaning that type II EC is associated with a high mortality rate [2, 6].

Molecular approaches to disease classification have led to the development of targeted therapies. These therapies, which can be broadly classified into angiogenesis inhibitors, tyrosine kinase inhibitors, PI3K/Akt/mTOR signaling modulators, human epidermal growth factor receptor (HER) antibodies, folate antagonists and dendritic cell immunotherapies, remain experimental for EC treatment and are typically reserved for patients for whom surgery has not been successful [2, 6]. There is therefore an urgent, unmet need for treatments that reduce the toxicities associated with current therapeutic approaches, improve patient outcome and reduce the reliance on surgical solutions to EC treatment.

In attempting to address the limitations of existing therapies, antibody-drug conjugates (ADCs) have emerged as a promising therapeutic approach that combines the selectivity of a targeted treatment with the cytotoxic potency of chemotherapy agents. The first ADC gemtuzumab ocogamicin (Mylotarg®) gained clinical approval in 2000 [10], paving the way for three further ADCs, brentuximab vedotin (Adectris®), ado-trastuzumab emtansine (Kadcyla®) and Inotuzumab ozogamicin (Besponsa®), which were licensed for the treatment of Hodgkin’s and anaplastic large-cell lymphomas, HER-2 positive breast cancer and relapsed or refractory B-cell precursor acute lymphoblastic leukemia, respectively [11–13].

An essential facet of ADC development is the selection of an appropriate target molecule that is specifically over expressed within cancerous tissue compared to normal tissue. Previous work within our laboratory has identified an association between the Receptor for Advanced Glycation End products (RAGE), a multi-ligand signaling system that drives innate immune inflammatory responses via NF-kB mediated gene activation, and gynecological disease [14]. Non-essential to life, RAGE expression in healthy tissue is absent or very low [15]. The only exception to this is the lungs, which express higher levels of unique RAGE isoforms not found elsewhere. Indeed, non-lung cells express RAGE mRNA that is up to three times the length of mRNA expressed in the lung and furthermore, the majority of cell lines studied, lack isoforms present in the lung [16]. Studies on the function of RAGE in murine knockout models and humans, suggest a homeostatic role in innate immunity, specifically related to the regulation of sepsis [15, 17]. Interestingly, ligand binding to RAGE does not facilitate clearance or degradation, but rather leads to a sustained period of receptor-mediated activation and RAGE over-expression. Over-expression and prolonged proinflammatory signaling are therefore associated with a number of diseases including Alzheimer’s, viral infections and the progression of several cancers [15, 18–20].

With the aim of developing a novel RAGE-targeting ADC, we describe in vitro and in vivo characterization with the goal of identifying lead candidates for pre-clinical development. Immunohistochemistry confirmed the over expression of RAGE in EC patients and thus the suitability of RAGE as a target molecule. Novel antibodies targeting different regions of the RAGE protein were characterized in vitro. Monoclonal selection based on antibody-peptide affinity, full characterization of antibody-antigen kinetics using Surface Plasmon Resonance, internalization dynamics, in vitro toxicity in cancer cell lines and in vivo bio-distribution and toxicology identified antibodies targeting the V-region of RAGE as suitable candidates for pre-clinical development. Our studies also confirm that RAGE-targeting ADCs are selectively toxic to RAGE expressing tumour cells in vitro, non-toxic to normal tissue/organs in vivo, and effectively reduce tumor growth in vivo.

## Materials and methods

Detailed methodologies relating to cell culture, antibody-drug conjugation, epitope mapping, surface plasmon resonance and gene and protein expression analysis are available as Additional file 1: supplemental methods. All cell lines were obtained from The European Collection of Authenticated Cell Cultures (ECACC, Public Health England, UK) between 2013 & 2015 and verified mycoplasma-free using the MycoAlert™ mycoplasma detection kit (Lonza, Castleford, UK). All experiments involving cell lines were conducted between passages 5 and 10 following thawing.

### Patient samples

Endometrial biopsies from 161 patients (70 control, 54 type I EC and 37 type II EC) were obtained from patients attending general gynecology clinics or postmenopausal bleeding (PMB) clinics within the Swansea Bay and Cwm Taf Morgannwg University Health Boards (SBUHB and CTMUHB). Postmenopausal patients that presented with bleeding or abnormally thickened endometrium (over 4 mm), identified incidentally in imaging investigations (abdominal ultrasound, MRI) performed for other clinical reasons, were included in the study. All patients with PMB or thickened endometrium underwent transvaginal ultrasound and Pipelle endometrial biopsy and hysteroscopy. Patients with cancer diagnosis at Pipelle biopsy underwent staging MRI and were scheduled for hysterectomy and/or bilateral salpingoophorectomy for type I disease, and hysterectomy, bilateral salpingoophorectomy, omentectomy and pelvic node dissection for type II disease. The control group included postmenopausal patients that underwent hysterectomy for vaginal prolapse and PMB patients with normal Pipelle sample and hysteroscopy findings.

Histological evaluation of endometrial samples, cancer diagnosis and staging was confirmed by the pathology department in SBUHB as part of routine clinical care. For endometrial cancer type I tumors included grade 1 and grade 2 endometrioid adenocarcinoma. Endometrial cancer type II included serous, clear cell and mixed adenocarcinoma tumors and high-grade endometrioid tumors (grade 3).

Follow-up time was up to 60 months. Survival was defined as the date from confirmed histological diagnosis after primary surgery to the date of death. Disease-free time was defined as the date from confirmed histological diagnosis after primary surgery to the date of recurrence or last visit (for those in the study for less than 60 months).

Patients that were peri or premenopausal, presenting with abnormal uterine bleeding (menorrhagia, intermenstrual bleed, postcoital bleed, amenorrhea) were excluded from this study. Patients with infection, chronic inflammation, autoimmune disease, endometritis, endometrial hyperplasia, and other cancers were excluded from the study. Ethical approval for immunohistochemistry analysis of FFPE EC patient samples within the study was obtained through Local Research Ethics Committee (reference 07/WMW02/50) for the collection of biopsies from consented EC patients (prospective analysis). Formal written consent was obtained from all patients at the time of recruitment into the study. Patients in control and study groups were matched with regard to body mass index and smoking status.

Data on age, BMI, parity, smoking status, menopausal status, hormone intake of any type, and comorbities were recorded in the study database. Data were also recorded on surgical procedure, histological type and stage, adjuvant treatment (radiotherapy, chemotherapy), follow up, recurrence free period, post-recurrence treatment and overall survival period.

### Antibody production

Monoclonal antibodies against RAGE were produced using standard protocols for monoclonal antibody production [21]. Briefly, mice were immunized with keyhole limpet hemocyanin (KLH)-conjugated RAGE, or KLH-conjugated peptides corresponding to amino acids (aa) 198–217 or 327–344 of the RAGE protein. Clones were selected based on a positive ELISA screen using bovine serum albumin (BSA)-conjugated peptides. Post-fusion, individual clones were selected by limiting dilution and clonal expansion to identify genetically stable, antibody producing cells for subsequent antibody production. One clone with affinity for the full RAGE protein (RBGO1), two clones with affinity for aa198–217 (RBGO2 and RBGO3) and one with affinity for aa327–344 (RBGO4) were selected for antibody production. Antibodies were purified from tissue culture medium using protein G affinity purification.

### Experimental design

#### RAGE expression in endometrial cancer and hyperplasia.

Endometrial biopsies were obtained from patients with a confirmed diagnosis of endometrial cancer (Type I, *n* = 54; Type II, *n* = 37), or endometrial cancer-free patients (control, *n* = 70). Preparation of formalin-fixed paraffin-embedded samples, nuclei staining, and immunohistochemistry was performed as previously described using a Ventana machine (Ventana Biotek Solutions, Tucson, AZ, USA) [22]. Positive (tonsil) and negative (endometrial tissue lacking antibody) control sections were used for reference. For immunohistochemistry, the anti-human RAGE antibody (RBGO1) was used. Slides were evaluated using a scoring system where slides are independently read by three observers (LM, NT, DG) on a multi headed microscope. The observers were blinded to the patients’ diagnosis, and demographics. The intensity of staining was scored from (0)-absent to (4)-strong. The distribution of staining was assessed as follows: (0)-absent, (1) - less than 30%, (2)-30 to 60%, (3)-more than 60% and (4)-100% of the tissue surface stained. The data was not normally distributed, the scoring results for the combined data of all the samples was analyzed using the Kruskal Wallace test followed by the Mann Whitney test.

#### RAGE expression in endometrial cancer cell lines

Endometrial cancer or normal endometrial cells were seeded (1 × 10^5^ cells/ml) in 6-well tissue culture plates (TPP, Trasadingan, Switzerland) in 2 ml of stripped medium, which comprised phenol red-free DMEM/F12 supplemented with 10% 2 x charcoal stripped FBS, 100 units/ml penicillin and 100 μg/ml streptomycin. Cells were cultured for 72 h in a humidified, 5% CO_2_ in air atmosphere at 37 °C. For *RAGE* mRNA analysis, supernatants were discarded and cells stored in RLT buffer (Qiagen) at − 80 °C prior to mRNA analysis by quantitative (q) PCR. For RAGE protein analysis, supernatants were discarded and cells stored in RIPA buffer at − 80 °C prior to total cell protein analysis by western blot.

#### Internalization of anti-RAGE antibodies

Endometrial cancer or non-malignant, primary endometrial stromal cells (ESC) were seeded (1 × 10^5^ cells/ml) in 8-well chamber slides (BD Biosciences, Oxford, UK) in 200 μl of stripped medium and cultured for 24 h in a humidified, 5% CO_2_ in air atmosphere incubator at 37 °C. After culture, cells were washed in pre-warmed (37 °C) Dulbecco’s phosphate buffered saline (DPBS) and slides placed on ice. Cells were treated with control medium or medium containing one of the α-RAGE antibodies at 10 μg/ml, and the 8-well chamber slides were incubated on ice for 30 min. Slides were then transferred to the incubator at 37 °C for 15, 30, 60, 120 or 240 min, before washing in DPBS and then fixing in 4% paraformaldehyde at 4 °C for 20 min. Where appropriate, cells were permeabilized following fixation, by incubation in 0.01% triton X-100 in DPBS at 4 °C for 10 min. Conjugation to the pHAb Amine Reactive Dye was done according to the manufacturer’s instructions (Promega, UK, Cat. No. G983). Cells were then washed and stained with goat anti-mouse IgG-Alexafluor488 diluted 1:1000 in DPBS before nucleus staining with DAPI. Images were acquired on a Zeiss LSM 710 confocal microscope (Carl Zeiss Microscopy, Jena, Germany), and analyzed using the Zen 2012 (blue edition) image analysis software (Carl Zeiss).

#### RAGE-ADC in vitro efficacy screening

##### For 2D screening:

Endometrial cancer or non-malignant, primary ESC were seeded (5 × 10^2^ cells/ml) in 96-well tissue culture plates (TPP) in 100 μl of stripped medium and cultured for 24 h in a humidified, 5% CO_2_ in air atmosphere incubator at 37 °C. After culture, cells were treated with control medium or medium containing ADCs (0.01–100 μg/ml), α-RAGE antibody (0.01–100 μg/ml), vcE (0.01–100 μM) or mcF (0.01–100 μM), for 96 h. Positive controls were cells treated with 0.01% Triton X-100 in stripped medium for the last 4 h of the experiment. Cell growth was monitored over the 96 h period using the RealTime-Glo™ MT Cell Viability Assay (Promega, Southampton, UK) in accordance with the manufacturer’s instructions. Fluorescence was measured at 24 h intervals using a FLUOstar Omega microplate reader (BMG Labtech, Aylesbury, UK).

##### For 3D screening:

Endometrial cancer cells were seeded (1 × 10^3^ cells/well) in a 96-well black ULA plate in 100 μl of stripped medium and cultured for 24 h in a humidified, 5% CO_2_ in air atmosphere incubator at 37 °C. After culture, cells were treated with control medium or medium containing RBGO1 ADC (0.01–100 μg/ml), RBGO1 antibody or mcF for 72 h. Cell viability was evaluated after 72 h using the CellTiter 3D Glo Viability Assay (Promega, Southampton, UK) in accordance with the manufacturer’s instructions. Luminescence was measured using a FLUOstar Omega microplate reader (BMG Labtech, Aylesbury, UK).

#### RAGE-ADC in vivo toxicity

In vivo toxicity studies were undertaken at Axis BioServices. All procedures were performed in accordance with the Animals (Scientific Procedures) Act 1986, and the guidance issued in *‘Responsibility in the case of Animals in Bioscience research: expectations of the major research council and charitable funding bodies.’*

Nude athymic mice, aged 5–7 weeks and weighing approximately 28-35 g, were divided into three treatment groups of six mice each. Mice were treated with PBS (control) or RBGO1 ADC at either 3 mg/kg or 20 mg/kg via intravenous injection. Bodyweight was measured at days 3, 6, 8, 13, 17 and 21 and mouse health assessed daily. Half of the mice in each group were sacrificed at 24 h and the remaining half 3 wks following dosing. After sacrifice, full blood counts were performed and serum aspartate aminotransferase (AST) activity assessed by ELISA, in accordance with the manufacturer’s instructions. Organs: brain, heart, lungs, stomach, pancreas, liver, kidneys, ovaries, uterus, bowel and spleen, were removed following sacrifice. Preparation of formalin-fixed paraffin-embedded samples was performed as previously described using a Ventana machine (Ventana Biotek Solutions, Tucson, AZ, USA) [22].

### HEC1A xenograft in vivo tumor reduction

All procedures were performed in accordance with the Animals (Scientific Procedures) Act 1986, and the guidance issued in *‘Responsibility in the case of Animals in Bioscience research: expectations of the major research council and charitable funding bodies.’*

Briefly, 6 week-old female athymic nude mice were subcutaneously inoculated with 5 × 10^5^ HEC1A cells. Mice bearing 5 mm in diameter tumours were distributed into three groups of 5 mice each. Mice were treated with control (PBS), RBGO1 ADC (3 mg/kg) or mcF (45 μg/kg, which is equivalent to the drug dose delivered by the ADC) via intravenous injection. Treatments were performed twice weekly for 4 weeks and tumour volumes were measured twice weekly. Tumours and organs: brain, heart, lungs, stomach, pancreas, liver, kidneys, ovaries, uterus, bowel and spleen, were removed following sacrifice. Preparation of formalin-fixed paraffin-embedded samples was performed as previously described using a Ventana machine (Ventana Biotek Solutions, Tucson, AZ, USA) [22].

### Statistical analyses

Statistical analyses were performed using IBM SPSS Statistics 22 with biological replicate as the experimental unit. Initially the data were tested for homogeneity, and log or square root transformed if appropriate. Parametric data were analyzed by analysis of variance (ANOVA) using Dunnett’s pairwise multiple comparison t-test for individual group comparisons. Non-parametric data were analyzed by Kruskal-Wallis followed by Mann Whitney U tests for multiple comparisons. Associations were analyzed using Factorial Logistic Regression. Overall survival and disease-free period was analyzed using Kaplan-Meier survival analysis and where appropriate, curves compared using the Log Rank (Mantel-Cox) test. Correlation within the patient data was determined using Spearman’s Rank Order Correlation. Co-localisation within the internalization experiments was determined using Pearson’s correlation coefficient (PCC) and Manders co-localisation coefficient. Data are presented as mean with standard deviation (SD), *p* < 0.05 was considered statistically significant, and the number of independent experiments is stated in the figure legends.

## Results

### The receptor for advanced glycation end products is over expressed in endometrial cancers and associated with reduced survival

Endometrial biopsies from 161 patients (70 control, 54 type I EC and 37 type II EC) were obtained as described in *Methods* (Patient demographics are shown in Additional file 2: Table S1). Median age at presentation was 57.5 ± 10.3, 67 ± 14.8, or 72 ± 6.0 years, respectively. Mean body mass index (BMI) at presentation was 31.1 ± 7.1, 35.6 ± 11.6, or 31.0 ± 6.2, respectively. Within the patient cohort age was a significant determining factor for EC (Factorial Logistic Regression = Type I EC: LR χ^2^ = 9.836, *p* = 0.003; Type II EC: LR χ^2^ = 25.229, *p* < 0.001), but BMI, smoking, parity and diabetes were not.

**Fig. 1:**
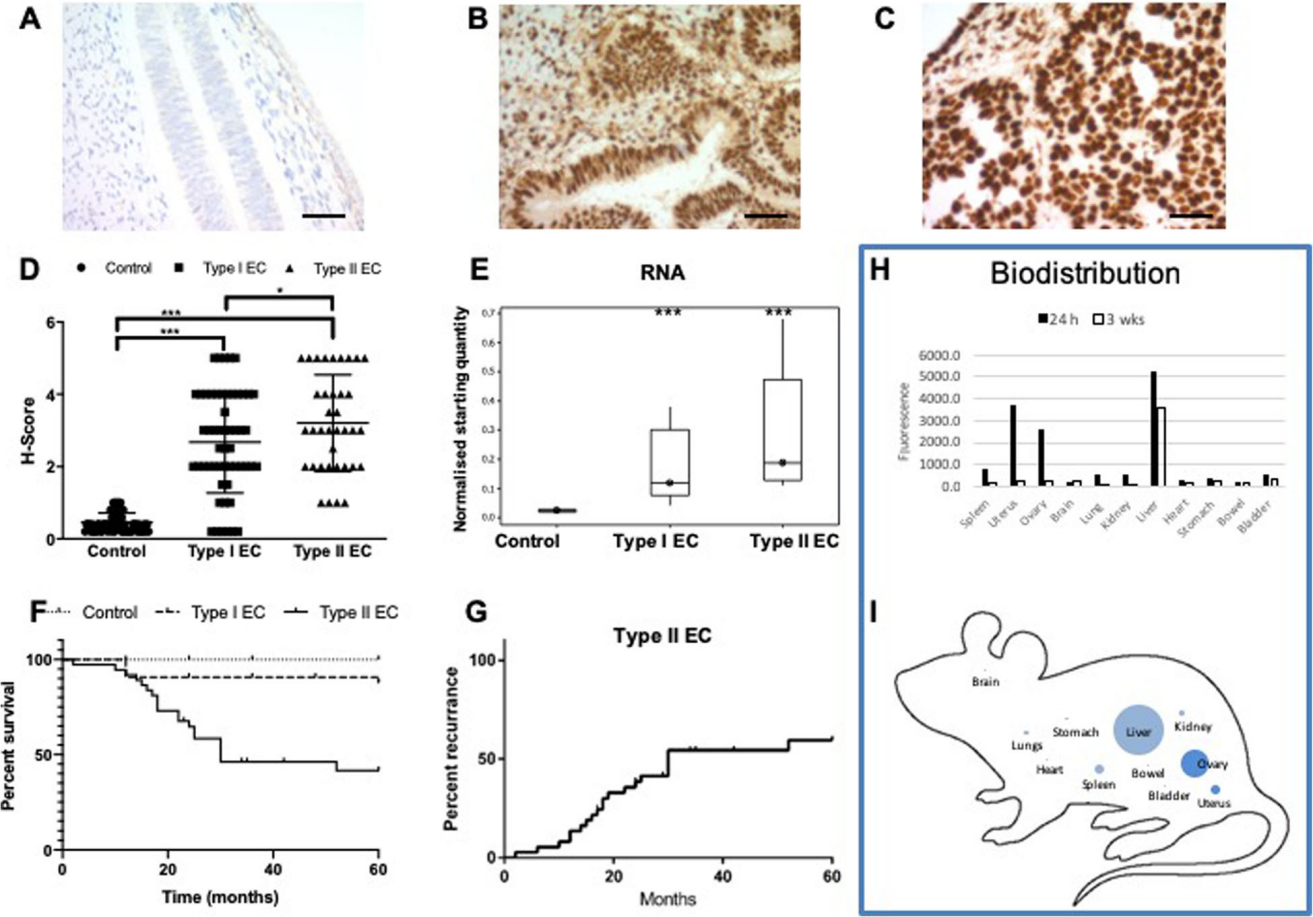
The receptor for advanced glycation end products (RAGE) is over expressed in endometrial cancer (EC) and associated with reduced survival. RAGE expression was determined by immunohistochemistry in biopsies (*n* = 67) from healthy patients (**a**; *n* = 25) and patients with type I (**b**; *n* = 24) or type II (**c**; *n* = 18) EC. Biopsies were formalin fixed and paraffin embedded before sectioning and staining with α-RAGE antibody. Representative images were acquired on a Zeiss Axio Imager 2 microscope and analyzed using the ZEN 2012 image analysis software. Scale bars are 50 μm. RAGE expression (H-score) was conducted blind by three of the authors (NT, LM & DG) independently and the mean score for each slide used (**d**). Kaplan-Meier survival curves were constructed using Graph Pad PRISM 6 based on survival (months) following surgery (**e**). Within type II EC patients, time to disease recurrence following surgery (months) was monitored (**f**) and correlated with RAGE expression (**g**). Biodistribution studies were perfomed in nude athymic mice, which were dosed intravenously with anti-RAGE antibody conjugated to the fluorophore Alexa-750 (3 mg/kg) and sacrificed after either 24 h or 3 wks. Organs were harvested and homogenized and the fluorescence from the tissue slurry measured using a fluorescence microplate reader (Varioskan LUX, ThermoFisher) at wavelength 750 nM. Fluorescence was normalised using the weight of the tissue and values expressed as Fluorescence Intensity per gram of tissue (**h** & **i**). Data points for RAGE expression (H-score) represent individual patients (**d**). Data were analyzed by ANOVA and Dunnett’s pairwise multiple comparison test; values differ from healthy, ***p < 0.001, *p < 0.05

RAGE expression was apparent in the stromal cells of the endometrium and was also detected in glandular/luminal epithelium. Expression within control endometrium was limited (Fig. 1a), whilst within endometrial biopsies from type I (Fig. 1b) or type II (Fig. 1c) EC, significant RAGE expression was observed. Semi-quantitative analysis (H-score) of RAGE expression in each of the patient groups showed a significant increase in RAGE expression in type I and type II EC compared to control patients (Fig. 1d; *p* < 0.001). RAGE expression was also signifncantly greater in the type II EC patient group compared to the type I EC group (Fig. 1d; *p* < 0.05). Additionally, quantification of RAGE mRNA in patient biopsies using quantitative (q) PCR, confirmed that RAGE mRNA expression was also significantly upregulated in type I and type II EC compared to control patients (Fig. 1e; *p* < 0.001). Kaplan-Meier overall survival analysis over a 5 y period, using number of months of survival following surgery, indicated a significantly reduced survival for type II EC compared to control patients (Fig. 1f: Log Rank (Mantel-Cox) test; *p* < 0.0001). Furthermore, correlation analysis showed a significant correlation between increased RAGE expression and reduced survival in the type II EC group (Spearman’s Rank Order Correlation: ρ = − 0.3914, *p* < 0.05, Additional file 3: Figure S1A). Disease recurrence following initial treatment was also common within the type II EC group (60% by 29 months; Fig. 1g). Correlation analysis again showed a significant correlation between increased RAGE expression and a reduced disease-free period (Spearman’s Rank Order Correlation: ρ = − 0.4915, *p* < 0.01, Additional file 3: Figure S1B). No correlations between RAGE expression and patient age, BMI, smoking, parity or diabetes were apparent in any of the patient groups.

### Anti-RAGE antibodies - therapeutic approach and in vivo biodistribution

The association between RAGE and EC led us to consider anti-RAGE antibodies as a potential therapeutic approach. To this end, we examined RAGE expression within four EC cell lines (Ishikawa - type I EC; and HEC1A, HEC1B, HEC50 - type II EC) by western blot, confocal microscopy and qPCR. In agreement with patient biopsies, high expression of RAGE was apparent in all four cell lines but absent in primary, non-malignant endometrial cells (Additional file 4: Figure S2A). Quantification of *RAGE* mRNA revealed the same pattern of expression, with significantly (*p* < 0.05) more *RAGE* mRNA present in EC cell lines compared to non-malignant primary endometrial cells (Additional file 4: Figure S2C). Immunofloursecence analysis revealed RAGE localizes at the cell membrane (Additional file 4: Figure S2B), and that the expression of RAGE in type II EC cell lines is higher than the type I EC cell line tested (Additional file 4: Figure S2D & E). Furthermore, we evaluated the expression of RAGE in a variety of human tissues (Brain, breast, kidney, liver, lung, lymph node, pancreas, spleen and uterus, Additional file 5: Figure S3). Western blot analysis confirmed that RAGE expression was absent or very low in these healthy tissues as previously reported [15]. The only exception to this was the lung tissue, which is known to express higher levels of unique RAGE isoforms not found elsewhere [16].

Next we considered the efficacy of anti-RAGE antibodies as an EC therapeutic by exploring the ability of commercially available anti-RAGE antibodies to effect cell killing in vitro. The EC cell lines Ishikawa, HEC1A, HEC1B and HEC50 were exposed to the following anti-RAGE antibodies (1 μg/ml to 100 μg/ml) for periods up to 96 h: N-16 (Santa Cruz Biotechnology, Cat. No sc-8230), A-9 (Santa Cruz Biotechnlogy, Cat. No. sc-365,154), ab37647 (Abcam, Cat. No. ab37647), MAB 5328 (Merck-Millipore, Cat. No. MAB5328), ab3611 (Abcam, Cat. No. ab3611) and MAB11451 (Bio-techne, Cat. No. MAB11451). None of the anti-RAGE antibodies tested had any effect on cell health (Data not shown).

The abcence of in vitro cell killing with anti-RAGE antibody alone led us to explore ADCs targeting RAGE as a potentially more effective therapeutic strategy. Using a small panel of antibodies (RBGO1–4) previously developed and characterized within our laboratory [23], we explored the suitability of ADCs as a therapeutic approach to the treatment of EC. Initially, we conducted in vivo bio-distribution experiments to demonstrate the feasibility of such an approach (Fig. 1). Anti-RAGE antibody raised against the whole RAGE protein (RBGO1), conjugated to the fluorophore Alexa-750 (3 mg/kg), was administered intravenously to female athymic nude mice and mice were sacrificed after either 24 h or 3 wks. Organs were harvested and homogenized with the fluorescence from the tissue slurry measured using a fluorescence microplate reader (Varioskan LUX, ThermoFisher) at wavelength 750 nM. Fluorescence was normalised using the weight of the tissue and values expressed as Fluorescence Intensity per gram of tissue. After 24 h, accumulation of anti-RAGE antibody was apparent primarily in the uterus, ovary and liver. Lower concentrations of antibody were noted in the spleen, lung and kidney and concentrations within other organs were at the limit of detection (Fig. 1h & i). After 3 wks, antibody concentrations within all organs, with the exception of the liver, were at base levels (Fig. 1h).

As previously described, antibodies within the panel were raised against the whole RAGE protein (RBGO1); the C1 domain peptide, aa198–217 (RBGO2 and RBGO3) and the transmembrane proximal region, aa327–344 (RBGO4) [23]. To identify the binding region of the RBGO1 antibody that was raised against the whole RAGE protein, we conducted epitope mapping using a peptide array of 404, 15aa peptides with a 14aa overlap. Arrays were probed with RBGO1 antibody at 1, 10 or 100 μg/ml for 16 h at 4 °C and spot intensities imaged using a LI-COR Odyssey imaging system. Analysis of the spot intensities indicated that the RBGO1 antibody bound with high affinity to a highly conserved region within the V-domain of the RAGE protein.

### Anti-RAGE antibodies with high binding affinity for rRAGE are rapidly internalized following receptor binding and trafficked to the endosomal compartment

**Fig. 2:**
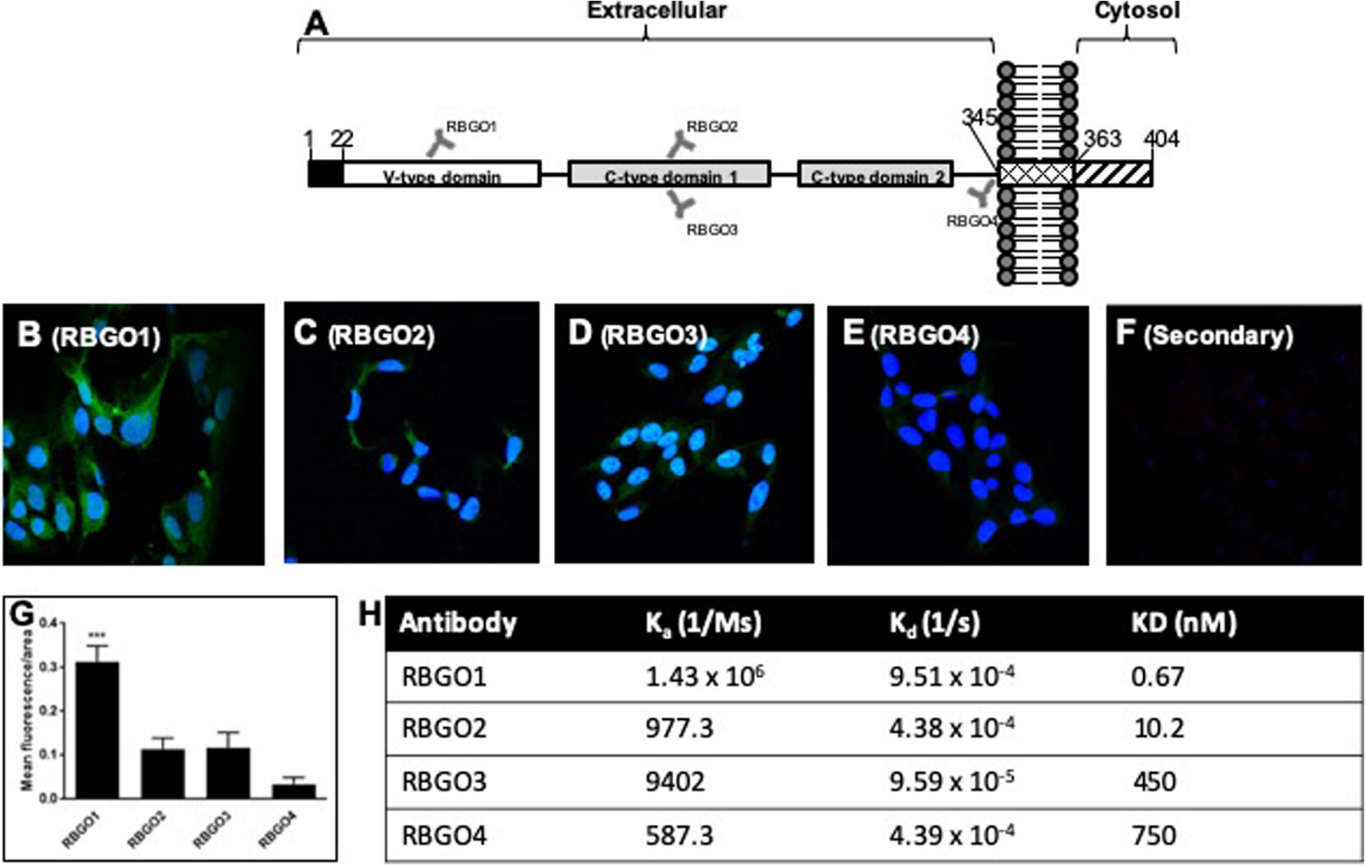
RBGO1 antibody, targeting the V-region of RAGE is internalized more rapidly than antibodies targeting other regions of the RAGE protein and binds with higher affinity to whole RAGE protein. Schematic diagram of the relative binding positions on the RAGE protein of each of the 4 antibodies tested (**a**). HEC1A endometrial cancer cells were treated with control medium or medium containing monoclonal antibodies against RAGE at 37 °C for 1 h. After incubation, the cells were washed, fixed and permeabilized. Internalized antibody: RBGO1 (**b**), RBGO2 (**c**), RBGO3 (**d**) or RBGO4 (**e**), was imaged via fluorescently labelled secondary antibodies and nuclei stained with DAPI. Cells were also incubated only with the secondary antibody as negative control (**f**). Images were acquired on a Zeiss LSM 710 confocal microscope and analyzed using the Zen 2012 image analysis software. The quantity of internalized antibody was determined using Image J software as a function of cell area (**g**). For antibody binding kinetics (**h**), antibodies were captured to a Sensor Chip CM5 via an amine coupled anti-mouse antibody followed by single-cycle kinetics experiments. RBGO1, RBGO2, RBGO3 or RBGO4 antibodies were exposed to whole RAGE protein (2.5 to 200 nM) and data were fitted using a one-to-one Langmuir binding model. Data are expressed as mean (SD) from 3 independent experiments. Data were analyzed by ANOVA and Dunnett’s multiple comparison test. RBGO1 differs from each of the other antibodies, ***p < 0.001.

Key to the development of an efficacious ADC is the internalization of antibody to facilitate cytotoxin delivery to the cell interior. Initial experiments assessed the internalization of our panel of antibodies in HEC1A cancer cells, which have high RAGE expression, following incubation with 1 μg/ml of each antibody over a 1 h period, using confocal microscopy (Fig. 2). Following fixing and permeabilization, staining with secondary antibody alone caused no non-specific binding or background fluorescence (Fig. 2f). HEC1A cells treated with the RBGO4 antibody (Fig. 2e) had the lowest fluorescence after 1 h, with approximately 2.5-fold more fluorescence in cells treated with RBGO2 (Fig. 2c) or RBGO3 (Fig. 2d) antibody, and approximately 7.5-fold more in cells treated with the RBGO1 antibody (Fig. 2b). Quantification of the mean fluorescence as a function of cell area, showed that the quantity of fluorescence in cells treated with the RBGO1 antibody was significantly more (*p* < 0.001; Fig. 2g) than for the other 3 antibodies. Although this pattern of internalisation matched our previous cell surface staining data [23], we evaluated antibody binding kinetics to whole RAGE protein via surface plasmon resonance (Fig. 2h). These data confirmed that, as previously, binding affinity between RBGO1 and RAGE was high, whilst binding to the other three antibodies was poor, thereby confirming that binding kinetics profile of this batch of antibodies was as previously described [23].

**Fig. 3:**
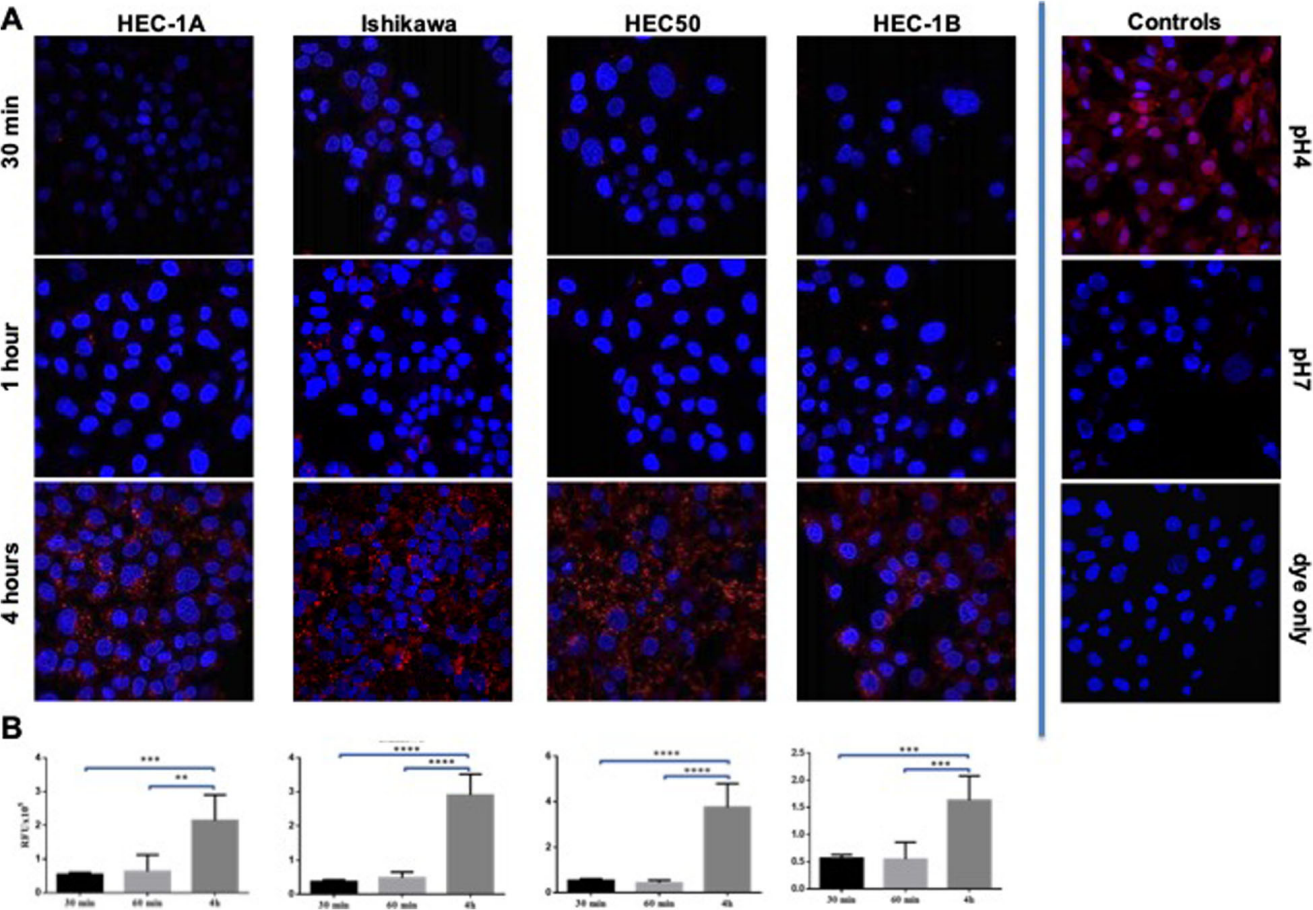
Conjugated RBGO1 antibody is rapidly internalized and trafficked to the endomsomal compartment. HEC1A, Ishikawa, HEC50 or HEC1B endometrial cancer cells were treated with control medium or medium containing RBGO1 antibody conjugated to a pH sensitive dye for 30 min, 1 h or 4 h (**a**). Images were acquired on a Zeiss LSM 710 confocal microscope and analyzed using the Zen 2012 image analysis software. The quantity of internalized antibody was determined using Image J software as a function of cell area (G). Histogram data are expressed as mean (SD) from 3 independent experiments (**b**). Data were analyzed by ANOVA and Dunnett’s multiple comparison test. Values differ from 30 min, **p < 0.01, ***p < 0.001, ****p < 0.0001

To confirm that the increased fluorescence observed was due to internalization and explore the mechanism of internalization, the RBGO1 antibody was conjugated to a pH sensitive dye, which fluoresces under low pH conditions (pH 6 to pH 4) [24]. As the pH found in endosomes and lysosomes falls within this range, tracking of the anti-RAGE antibody to these organelles is therefore possible once internalisation begins. Internalisation was measured in all four EC cell lines following incubation with the RBGO1-pH dye conjugate for 30 min, 1 h or 4 h (Fig. 3). Fluoresence imaging in all four EC cell lines (Fig. 3a) revealed the presence of internalized antibody as early as 30 min, followed by a significant increase in internalization up to 4 h. Quantification of this image data using the imageJ JavaScript confirmed the significant increase in internalized antibody over time (Fig. 3b). Additionally, these data indicate that conjugation of the anti-RAGE antibody does not impair internalization and thus its suitability for ADC development.

**Fig. 4:**
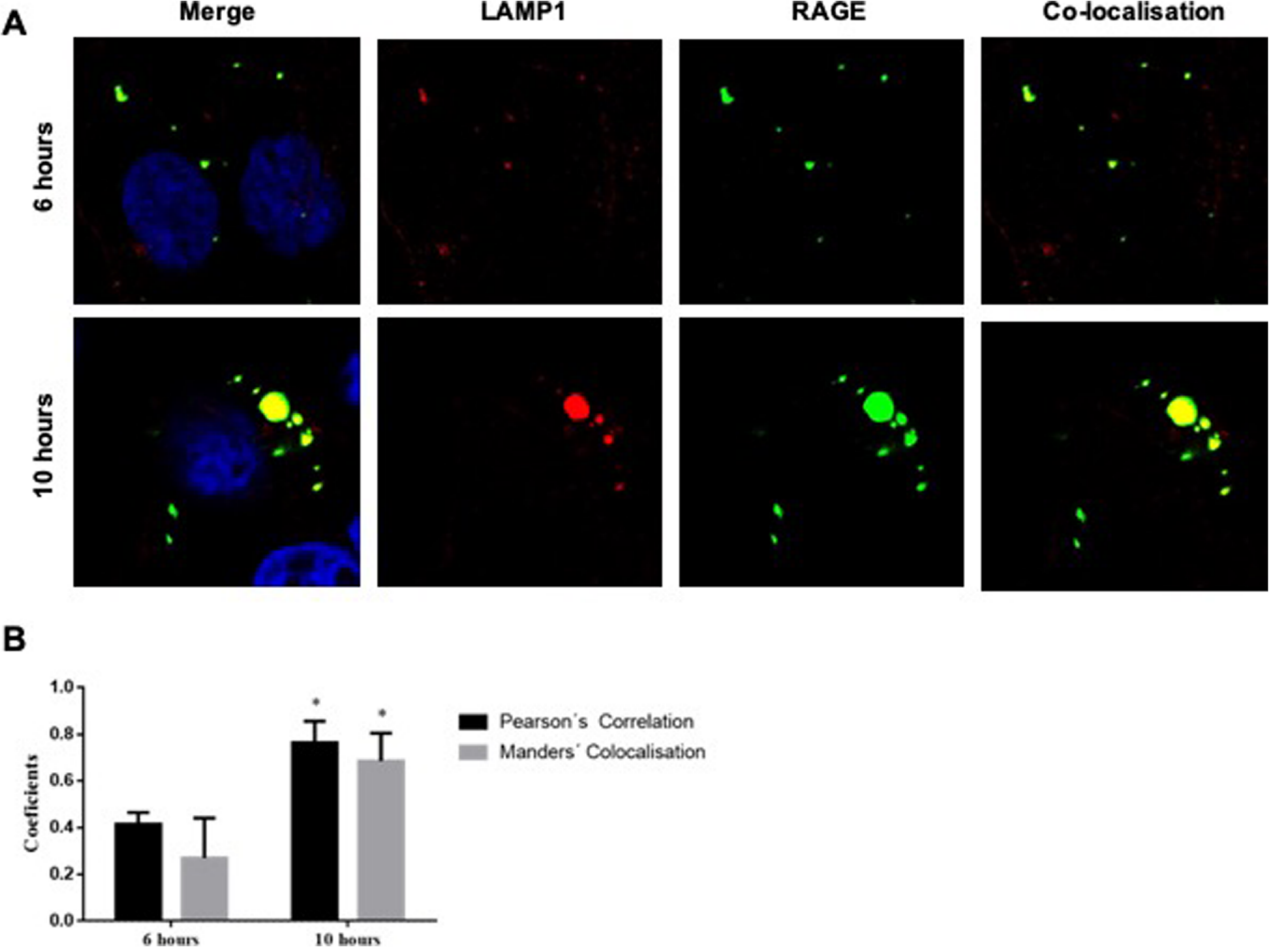
Conjugated RBGO1 antibody co-localises with LAMP1 indicating lysosomal trafficking and accumulation following internalization. Following inhibition of lysosomal action, HEC1A endometrial cancer cells were incubated with control medium or medium containing RBGO1 antibody conjugated to FITC (50 μg/ml) for 6 or 10 h. After fixing and permeablisation, cells were stained with a rabbit anti-LAMP1 antibody to detect the lysosomal compartment. Images were acquired on a Zeiss LSM 710 confocal microscope and analyzed using the Zen 2012 image analysis software (**a**). The quantity of internalized antibody was determined using Image J software as a function of cell area (G). Histogram data are expressed as mean (SD) from 3 independent experiments (**b**). Data were analyzed by ANOVA and Dunnett’s multiple comparison test. Pearson’s correlation coefficient (PCC) and Manders col-localisation coefficient were performed to assess co-localisation. Values differ from 6 h, *p < 0.05

Next we performed co-localisation experiments within the HEC1A EC cell line to assess lysomal transport and the accumulation of anti-RAGE antibodies following internalization (Fig. 4). Lysosomal action was first inhibited by incubation of the cells with 100 nM leupeptin for 1 h to prevent antibody degradation within the lysosome. Cells were then treated with anti-RAGE antibody conjugated to FITC (50 μg/ml) for 6 or 10 h. After fixing and permeablisation, cells were stained with a rabbit anti-LAMP1 antibody to detect the lysosomal compartment. Co-localisation of the RAGE and LAMP1 was apparent following 6 or 10 h of antibody exposure (Fig. 4a). The quantitative evaluation of co-localisation is required to confirm fluorophore overlap is not random. We therefore undertook Pearson’s correlation coefficient (PCC) and Manders col-localisation coefficient to assess co-localisation [25, 26]. Both methods confirmed the co-localisation of anti-RAGE and anti-LAMP1 antibodies, with up to 69% of internalized antibody located within the lysosomal compartment (Fig. 4b).

### Development and characterization of novel antibody-drug conjugates

To further explore the utility of a RAGE targeted ADC, we conjugated each of the four antibodies to the antimitotic agents: monomethyl auristatin E (MMAE), via a lysosomally cleavable dipeptide valine-citrulline (vc) linker; or monomethyl auristatin F (MMAF), via a non-cleavable maleimido caproyl (mc) linker (Additional file 6: Figure S4).

Drug loading of the conjugates was analyzed using a combination of hydrophobic interaction chromatography (HIC) and reverse phase chromatography - Polymer Laboratories Reverse Phase PLRP (Additional file 7: Figure S5). Due to the complex disulfide structure of an IgG2b antibody and potential conjugation site variability, the PLRP chromatographic patterns for the RBGO1 antibody (Additional file 7: Figure S5A, B) were too complex to accurately determine the average drug-antibody ratio (DAR). They did however indicate a good level of drug conjugation and analysis of the traces (Area Under Curve) suggested an average DAR of 3.5. For the RBGO2 (Additional file 7: Figure S5C, D), RBGO3 (Additional file 7: Figure S5E, F) and RBGO4 (Additional file 7: Figure S5G, H) antibodies, which were IgG1, the PLRP traces for both vcE and mcF were clearly discernible, showing drug loading up to a DAR of 4.

### Anti-RAGE ADCs preferentially kill endometrial cancer cells

**Fig. 5:**
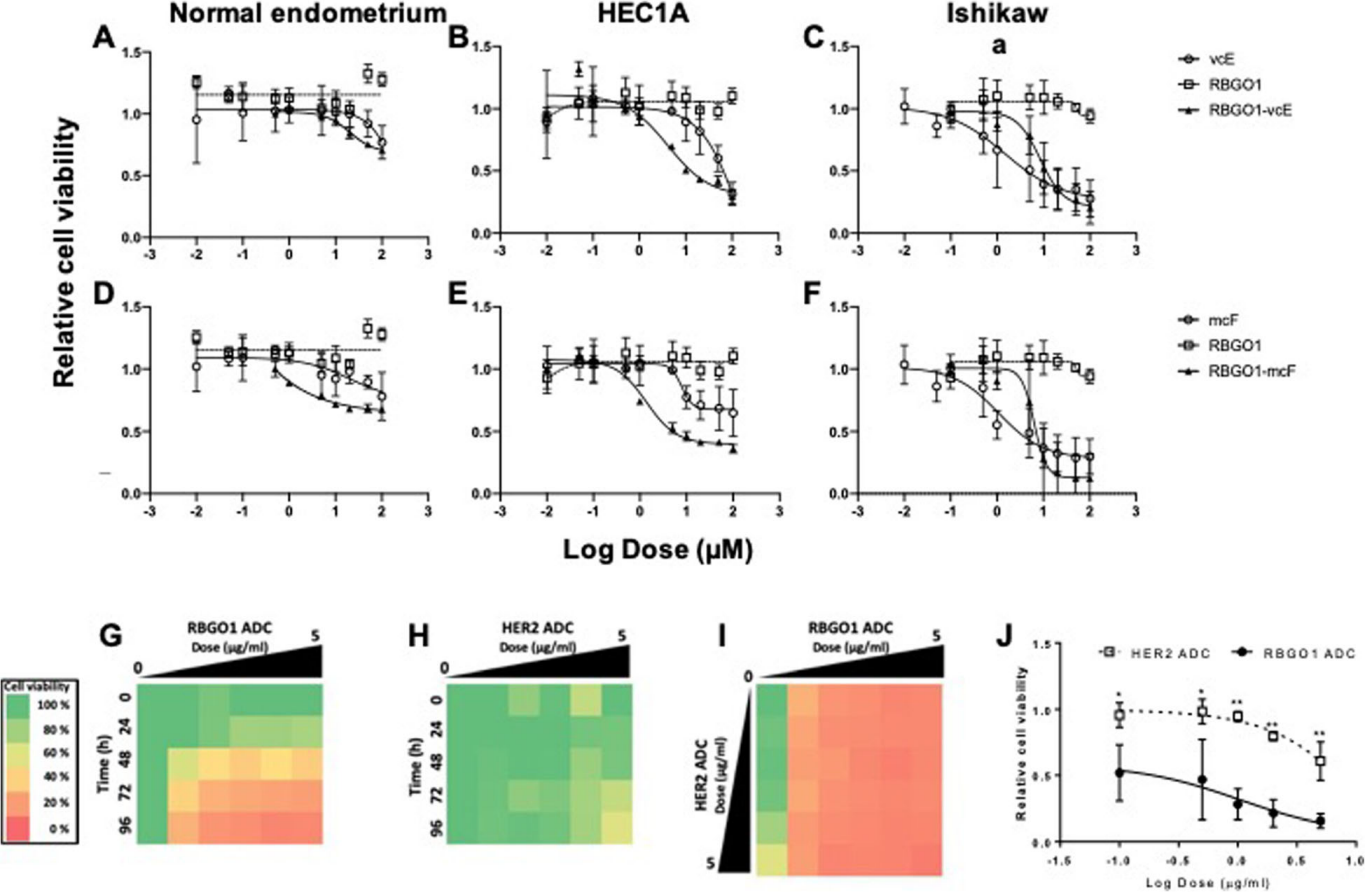
**a**–**f** RBGO1 ADCs preferentially target endometrial cancer cells and increase drug sensitivity by up to 200-fold. Normal endometrial, HEC1A cancer or Ishikawa cancer cells were incubated with control medium or medium containing vcE (**a**–**c**; 0.01 to 100 μM) or mcF (**d**–**f**; 0.01 to 100 μM), or RBGO1 (**a**–**f**), RBGO1 -vcE (**a**–**c**) or RBGO1 -mcF (**d**–**f**; 0.01 to 100 μg/ml) for 96 h. Cell viability was determined by RealTime-Glo™ MT Cell Viability Assay and lethal dose 50 (LD_50_) values determined following curve fitting using a 4-parameter logistic model. Drug equivalencies were calculated based on an average DAR of 3. Data are expressed as mean (SD) from 4 independent experiments and normalized to the untreated control to account for cell growth during the period of the experiment. (**g**–**j**) RBGO1 ADC is more efficacious in HEC1A EC cells than HER2 ADC. HEC1A EC cells were treated with RBGO1 ADC (**g**; 0.1 to 5 μg/ml), HER2 ADC (**h**; 0.1 to 5 μg/ml), or RBGO1 ADC and HER2 ADC (**i**; 0.1 to 5 μg/ml) for 96 h and cell viability determined at 0, 24, 48, 72 and 96 h using the RealTime-Glo™ MT Cell Viability Assay. Heat map color intensities were based on percent cell viability compared to the untreated control (**g**–**i**; see scale in figure). Relative cell viability plots were fitted using a 4-parameter logistic model (**j**). Data displayed are means of three independent experiments. Data were analyzed by ANOVA and Dunnett’s multiple comparison test. ADCs differs from each other within the same dose, *p < 0.05, **p < 0.01

We next compared cytotoxicity following the exposure of normal endometrial, HEC1A or Ishikawa cancer cells to antibodies, auristatins or ADCs (Fig. 5a–f). Cells were cultured in the presence of vcE (Fig. 5a–c; 0.01 to 100 μM), mcF (Fig. 5d–f; 0.01 to 100 μM), RBGO1 (Fig. 5a–f; 0.01 to 100 μg/ml), RBGO1-vcE (Fig. 5a–c; 0.01 to 100 μg/ml) or RBGO1-mcF (Fig. 5d–f; 0.01 to 100 μg/ml) for 96 h and cell viability determined using the RealTime-Glo™ MT Cell Viability Assay. Normal endometrial cells were resistant to killing by any of the treatments, with the lethal dose 50 (LD_50_) concentrations for all treatments being > 100 μM (Fig. 5a, d). LD_50_ values for HEC1A cells (Fig. 5b, e) were: vcE = 65 μM, mcF and RBGO1 > 100 μM, RBGO1-vcE = 13 μg/ml (≡ to 0.3 μM vcE) and RBGO1-mcF = 5 μg/ml (≡ 0.09 μM mcF). LD_50_ values for Ishikawa cells (Fig. 5c, f) were: vcE = 4 μM, mcF = 3 μM, RBGO1 > 100 μM, RBGO1-vcE = 11 μg/ml (≡ to 0.2 μM vcE) and RBGO1-mcF = 7 μg/ml (≡ 0.1 μM mcF). These data suggested that RBGO1-ADCs preferentially kill endometrial cancer cells compared to normal endometrial cells. Additionally, in Ishikawa cells, we observed a 20-fold increase in sensitivity to killing for RBGO1-vcE compared to vcE (LD_50_: 4 → 0.2 μM; Fig. 5l, c); and a 30-fold increase in sensitivity to killing for RBGO1-mcF compared to mcF (LD_50_: 3 → 0.1 μM; Fig. 4o, f). In HEC1A cells, we observed a more than 200-fold increase in cell sensitivity to killing for RBGO1-vcE compared to vcE (LD_50_: 66 → 0.3 μM; Fig. 4k, b), which could be due to higher RAGE expression in HEC1A cells compared to Ishikawa cells.

The similarity of the LD_50_ values between HEC1A and Ishikawa cells, led us to continue cytotoxicity testing in HEC1A cells only. We evaluated the cytotoxicity of RBGO2, RBGO3 and RBGO4 antibodies (0.01 to 100 μg/ml), and their respective ADCs (0.01 to 100 μg/ml) in normal endometrial and HEC1A cancer cells (Additional file 8: Figure S6. LD_50_ values were > 100 μM within normal endometrial cells for RBGO2 (Additional file 8: Figure S6A-C), RBGO3 (Additional file 8: Figure S6G-I) or RBGO4 (Additional file 8: Figure S6M-O) antibodies or ADCs. Within HEC1A cancer cells (Additional file 8: Figure S6D-F; RBGO2, J-L; RBGO3, P-R; RBGO4), LD_50_ values for RBGO2, RBGO2-mcF, RBGO3, RBGO3-mcF and RBGO4 were also > 100 μM. LD_50_ values for RBGO2-vcE, RBGO3-vcE, RBGO4-vcE and RBGO4-mcF were 95 μg/ml (≡ to 2 μM vcE), 70 μg/ml (≡ to 1.5 μM vcE), 116 μg/ml (≡ to 2.4 μM vcE) and 104 μg/ml (≡ to 2.2 μM mcF), respectively. These data suggested that ADCs comprising RBGO2, RBGO3 or RBGO4 antibodies were less efficacious than the RBGO1-ADC, which was at least 5- (RBGO2-vcE vs RBGO1vcE) to 24-fold (RBGO4-mcF vs RBGO1-mcF) more effective at killing HEC1A cancer cells.

Finally, to confirm that the RBGO1-ADC was specific for RAGE, we performed blocking experminents using a commercially available goat anti-human RAGE antibody (N-16, Santa Cruz Biotechnology, Cat. No sc-8230) and an anti-mouse Alexa 488 secondary, which would only bind to the RBGO1 antibody (Additional file 9: Figure S7). Ishikawa cells, prepared as previously described for the internalisation experiments, were fixed and stained for RAGE expression using the RBGO1 antibody, which produced the same pattern of RAGE staining seen previously (Additional file 9: Figure S7A). In contrast, pre-incubation of the cells for 1 h with the N16 antibody, followed by staining with the RBGO1 antibody showed no staining of the cells (Additional file 8: Figure S6B). These data confirm the specificity of the RBGO1 antibody for RAGE.

### RBGO1 ADC is more effective than a HER2 ADC at killing EC cells

Having determined the greater efficacy of the RBGO1 ADC compared to the other RAGE targeting ADCs. We evaluated the effectiveness of the RBGO1 ADC against a vcE conjugated ADC targeting the human epidermal growth factor receptor 2 (HER2), since this antigen is already used as a therapeutic target for the ADC, Kadcyla® (Fig. 5g–j). Peptide growth factors frequently implicated in EC include members of the type I receptor tyrosine kinase family, which includes HER2 [27]. Since over expression of HER2 is typically associated with type II EC [28, 29], we used HEC1A cells for our ADC comparison experiments because they are derived from a type II EC tumor and express high levels of HER2 [30]. Cells were cultured in the presence of RBGO1 ADC (Fig. 5g; 0.1 to 5 μg/ml), HER2 ADC (Fig. 5h; 0.1 to 5 μg/ml), or RBGO1 ADC and HER2 ADC (Fig. 5i; 0.1 to 5 μg/ml) for 96 h and cell viability determined at 0, 24, 48, 72 and 96 h using the RealTime-Glo™ MT Cell Viability Assay. The effectiveness of the RBGO1 ADC (Fig. 5g) in HEC1A cells was confirmed, with dosage and time-responses observed. A dose and time effect was also apparent for the HER2 ADC (Fig. 5h), although far less HEC1A cell killing was observed compared to the RBGO1 ADC. A combination therapy approach (Fig. 5i) demonstrated that after 96 h treatment with both ADCs, the contribution to HEC1A cell killing of HER2 ADC was minimal compared to the effect of RBGO1 ADC. This was further confirmed by statistical analysis of each of the doses tested at the 96 h time point (Fig. 5j), which demonstrated significantly more HEC1A cell killing by RBGO1 ADC compared to HER2 ADC (*p* < 0.05).

### RBGO1 ADC is not toxic in a murine in vivo model

To verify the suitability of RBGO1 ADC for full in vivo evaluation, we administered (intravenously) RBGO1 ADC (at 3 mg/kg or 20 mg/kg) to female, athymic mice. Bodyweight was measured at days 3, 6, 8, 13, 17 and 21 and mice were sacrificed at either 24 h or 3 wks following dosing, after which full blood counts and an aspartate aminotransferase (AST) ELISA were performed.

**Fig. 6:**
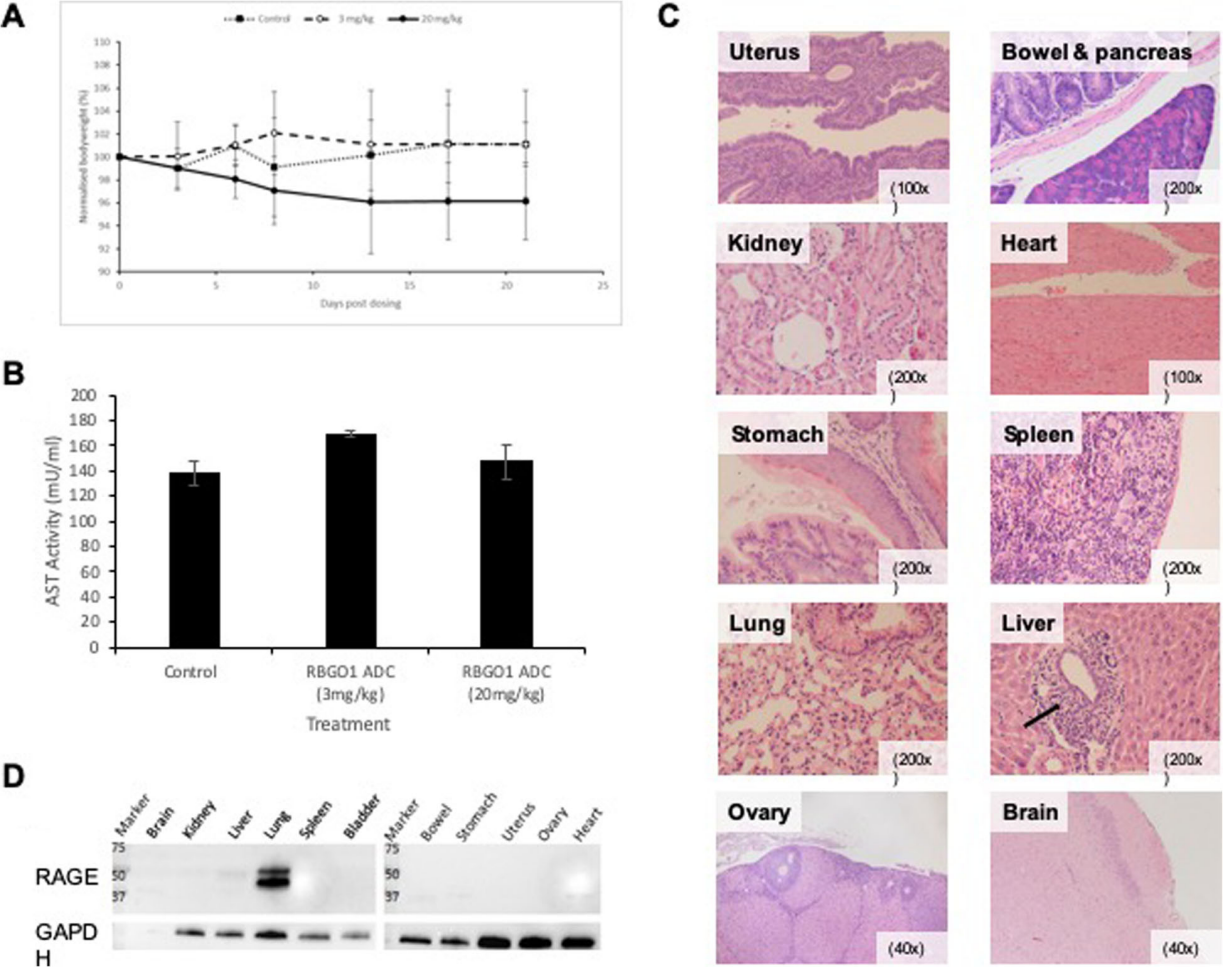
RBGO1 ADC is not toxic in a murine in vivo model. PBS (Control) or RBGO1 ADC was administered (intravenously) to female, athymic mice aged 5–7 weeks and weighing approximately 28-35 g, at a dose of either 3 mg/kg or 20 mg/kg. Bodyweight **a** was measured at days 3, 6, 8, 13, 17 and 21 and mice were sacrificed at either 24 h or 3 wks following dosing, after which full blood counts and an aspartate aminotransferase (AST) ELISA were performed (**b**). Organs were harvested immediately following sacrifice, and formalin fixed and paraffin embedded before sectioning and staining with hematoxylin and eosin (**c**). Western blot analysis of mouse tissue was performed using the RBGO1 antibody (**d**). Representative images were acquired on a Zeiss Axio Imager 2 microscope and analyzed using the ZEN 2012 image analysis software and magnifications are shown on each image. Low level inflammatory cell infiltration is indicated in the ‘*Liver’* image (⟶). Data displayed in histograms are means of three animals. Data were analyzed by ANOVA and Dunnett’s multiple comparison test. ADC treatments differ from control, **p < 0.01

Although bodyweight in animals treated with the high dose of RBGO1 ADC decreased slightly during the study, no significant changes were apparent (Fig. 6a). Full blood counts (Additional file 10: Table S2) indicated that animals treated with RBGO1 ADC (3 mg/kg) had a reduced white blood cell count compared to control animals. Animals treated with RBGO1 ADC (20 mg/kg) had reduced white blood cells and reticulocytes, and an increased platelet count compared to control animals. Serum AST activity was not elevated in any of the treatment groups 24 h after dosing and only in the RBGO1 ADC (20 mg/kg) treatment group 3 wks after dosing (Fig. 6b). However, no signs of distress or ill health were noted during the study in any of the treatment groups, indicating that any toxicity caused by the RBGO1 ADC was minimal even in the high dose treatment group. Furthermore, histological analysis (Fig. 6jc) demonstrated an absence of toxicity across all the treatment groups. A low level of inflammation was noted in the liver, lungs and kidneys of some animals, but as this was observed in all treatment groups including the control it was not a consequence of treatment with RAGE-ADC (Additional file 11: Table S3). Cross-reactivity of the RBGO1 antibody with murine RAGE was confirmed by western blot analysis using the RBGO1 antibody (Fig. 6d). RAGE expression was absent in brain, kidney, spleen, bladder, bowel, stomach, uterus, ovary and heart, with weak expression in the liver and high expression in the lungs noted.

### RBGO1 ADC reduces tumor volume in a murine xenograft model

**Fig. 7:**
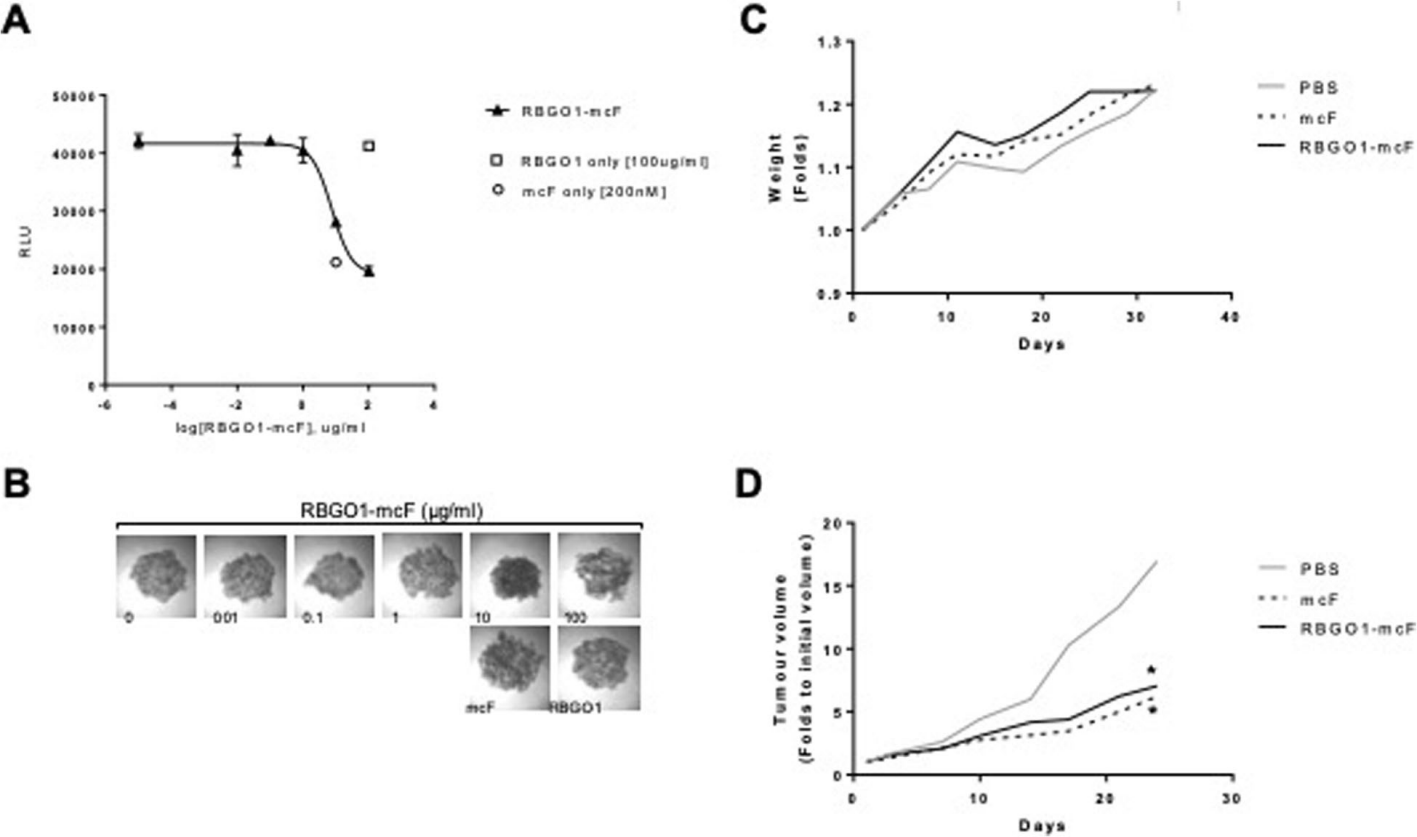
RBGO1 ADC is effective within a 3D in vitro tumour model and effectively reduces tumour growth in a murine xenograft model of disease. **a**, **b** HEC1A cells cultured in low-adherent plates to enable spheroid formation, were incunbated with medium containing RBGO1 ADC (0.01–100 μg/ml), RBGO1 antibody (100 μg/ml) or mcF (200 nM) for 72 h. Cell viability was determined using the CellTiter 3D Glo Viability Assay and luminescence measured using a FLUOstar Omega microplate reader. Representative images of spheroids were acquired on a Zeiss Axio Imager 2 microscope. Relative cell viability plots were fitted using a 4-parameter logistic model (J). Data displayed are means of three independent experiments. **c**, **d** RBGO1 ADC (3 mg/kg), mcF (45 μg/kg) or PBS (Control) were adminstered intravenously to female athymic, nude mice bearing 5 mm HEC1A xenograft tumours on a twice weekly basis for 4 weeks. Bodyweights and tumour volumes were measured twice weekly. After 4 weeks, mice were sacrificed and organs harvested for evaluation of any systemic toxicity. Data displayed in are means of five animals with error bars omitted for clarity. Data were analyzed by ANOVA and Dunnett’s multiple comparison test. Treatments differ from control (PBS), *p < 0.05

To evaluate the efficacy of RBGO1 ADC in vivo we first explored the utility of the ADC within a 3D culture model (Fig. 7a, b). HEC1A cells were cultured in low adherent culture plates to enable the formation of spheroids. Once formed, spheroids were treated with RBGO1 ADC (0.01–100 μg/ml), RBGO1 antibody (100 μg/ml) or mcF (200 nM) for 72 h. After treatment, cell viability was evaluated using the CellTiter 3D Glo Viability Assay. As with the 2D culture cell killing experiments, treatment with RBGO1 antibody had no observable effect on cell viability, whilst treatment with mcF was effective. The LD_50_ for RBGO1 ADC was 7.4 μg/ml, which was similar to that noted within the 2D culture experiments and confirmed the potential for the RBGO1 ADC to be effective in vivo.

In vivo efficacy was evaluated by administering RBGO1 ADC (3 mg/kg) or mcF (45 μg/kg) intravenously to female athymic, nude mice bearing 5 mm HEC1A xenograft tumours on a twice weekly basis for 4 weeks. Bodyweights and tumour volumes were measured twice weekly. After 4 weeks, mice were sacrificed and organs harvested for evaluation of any systemic toxicity.

In keeping with the single dose toxicity studies, no toxicity was observed in any organs for either treatment (data not shown). No significant changes in bodyweight were apparent (Fig. 7c). Treatment with RBGO1 ADC or mcF, however, significantly reduced the rate of tumour growth compared to the PBS control (Fig. 7d, *p* < 0.05) suggesting the suitability of the RBGO1 ADC as a potential therapeutic approach.

## Discussion

An increasing incidence of gynecological cancer [1], together with continuing problems with toxic side-effects of current cytotoxic therapies, mean new strategies are needed to address the treatment challenges posed by varying chemotherapy responsiveness and chemotherapy-resistant populations [31]. This study explored the targeting of a novel biomarker of EC, RAGE, with ADCs to determine the suitability of this therapeutic strategy for EC.

Differential RAGE expression between non-malignant and malignant endometrial cells was observed in patient biopsies and the cell lines used within this study. Elevated RAGE mRNA and intensive RAGE staining was evident in biopsies from patients with type I or type II EC, whilst expression in healthy patients was minimal. RAGE was also clearly discernible by western blot, confocal microscopy and PCR in EC cell lines, but absent in non-malignant primary endometrial cells from patients and all major tissues. Additionally, increased RAGE expression was correlated with a reduced disease-free survival time in patients with type I or type II EC, confirming an association between RAGE and EC. In this context, several clinical studies have described a strong association between RAGE expression and the aggressiveness of various cancer types [32]. Indeed, the clear association between cancer and RAGE expression and function, is well documented with reports demonstrating RAGE expression associated with breast cancer, gastric cancer, colon cancer, hepatocellular carcinoma, pancreatic cancer, prostate cancer, ovarian cancer and lung cancer, among others [33–39]. It is also noteworthy that RAGE expression is only reduced in lung cancer, suggesting RAGE may act as a tumour suppressor in this organ [32]. Data obtained from murine cancer models supports a mechanistic role for RAGE activation whereby induction of cell signaling proteins such as AKT proteins, the anti-apoptotic protein, BCL2, and cyclin D1, promote tumor cell proliferation. Additionally, RAGE activation limits apoptosis by inhibiting translocation of pro-apoptotic p53 to the mitochondria and enhancing tumor cell mitochondrial complex I activity and ATP production, thereby changing the bioenergetics of the cells to support tumor growth [19, 32, 40, 41].

Within our data, high RAGE expression in EC was correlated with a poor disease-free, or overall survival time, and expression in type II EC was higher than in type I EC suggesting an association with tumor aggressiveness in our patient cohort.

RAGE also provides a link between inflammation and cancer development. By inducing and sustaining a pool of transcriptionally active NF-κB proteins, RAGE signaling maintains an inflammatory environment that drives cancer progression. Thus, RAGE seems an appropriate target for the development of novel therapies for treating epithelial malignancies, including EC. Indeed, anti-RAGE antibodies have previously been evaluated in murine models for the treatment of acute sepsis (XT-M4, a monoclonal antibody recognizing the V-domain of RAGE) [42]; halting endotoxemia-related organ disorders (abRAGE recognizing an epitope within the RAGE extracellular domains) [43]; the inhibition of peritoneal fibrosis in diabetic animals (anti-RAGE monoclonal antibody recognizing the RAGE extracellular domains) [44]; and the inhibition of tumor growth in a xenograph melanoma model (anti-RAGE polyclonal antibody recognizing the C1-domain of RAGE) [45].

Differential RAGE expression between non-malignant and malignant endometrial cells was sufficient to afford protection in vitro against RAGE targeting ADCs within non-malignant cells. At the highest dose of ADC investigated (100 μg/ml), the maximum killing achieved in non-malignant cells was 30%, whilst equivalent cell killing in EC cells was achieved at 1 μg/ml of ADC, meaning RAGE targeting ADCs were up to 100-fold more efficacious in EC cells compared to non-malignant cells. In EC cells cultured in 2D or 3D (spheroids), treatment with antibodies alone had no cytotoxic effect and treatment with auristatins alone had limited efficacy. In contrast, ADCs were up to 200-fold more efficacious than treatment with auristatin alone. Additionally, we compared the RBGO1 ADC to a comparable vcE-conjugated HER2 ADC, since this antigen is used as a therapeutic target for the ADC, Kadcyla®, HER2 is associated with EC, and is overexpressed in HEC1A cells [27–30, 46]. Importantly, the RAGE targeting RBGO1 ADC was more efficacious than the similar ADC targeting HER2. These data imply that the use of RAGE targeting ADCs as a therapeutic strategy is highly efficacious.

Key to the development of ADCs is the optimization of each constituent part [47]. We noted significant variability in the internalization of each of the four antibodies tested, which was associated with varying cytotoxic efficacy in EC cells. Several possible explanations for this variability presented themselves, including the location of antibody binding to RAGE protein. The main structural and functional unit for ligand binding is formed from the V- and C1 (VC1) regions of the RAGE protein, and the vast majority of RAGE ligands bind to this unit [48, 49]. Whilst a small number of RAGE ligands, such as S100A6 and lysophosphatidic acid, bind with low-affinity binding to the C2-domain [50, 51], even these bind with much greater affinity to the V-domain [52]. The VC1 region also drives the self-association of membrane-bound RAGE molecules in the absence of ligand [48], which is required for activation and downstream signaling [52]. It is therefore plausible that antibody binding location could influence receptor activation and internalization. Internalization is a well-known mechanism to shut down signaling of an active receptor/ligand complex [52]. Indeed, the rapid internalization of RAGE/S100 protein complexes into granular structures has been shown [53]. RAGE receptor activation following antibody binding, might therefore be an essential consideration when designing RAGE-targeting ADCs.

The V-region binding antibody, RBGO1, bound to rRAGE with greater affinity than the other antibodies tested and was associated with rapid internalization, tracking to the lysosomal compartment and greater cytotoxicity in vitro, implying the importance of this aspect of ADC design for RAGE targeting and indeed, potentially when targeting other membrane receptors for cancer therapy.

Preferential ligand binding through the VC1-domain occurs because the positive charge of this domain enables recognition of negatively charged ligands, even when the electrostatic signal is weak [54]. Under physiological conditions (pH 7) antibodies also carry an overall negative charge providing a rationale for the improved binding of the RBGO1 antibody and other antibodies targeting the V-domain, such as XT-M4 [42]. Additionally, the oligomerization driven by the VC1-domain produces clusters of RAGE molecules on the cell surface that bind ligands more strongly than single molecules of RAGE, and are important for sustained signaling [55]. By comparison, HER2 receptor clustering promotes the internalization of anti-HER2 antibodies [56]. Therefore, it is not surprising that rapid internalization of RAGE antibodies was observed in high RAGE expressing EC cells used in this study.

Since the bio-distribution studies had demonstrated a wide dissemination of anti-RAGE where some accumulation was observed in the liver, uterus, ovary and spleen, it was important to demonstrate the absence of any toxicity within the host. Initial evaluation of the toxicity of RAGE ADC in vivo indicted no significant toxicity associated with the any of the doses used. It is noteworthy that the high dose used (20 mg/kg) is three times higher than the high dose (7 mg/kg) typically used to determine maximum tolerated doses for ADCs clinically [57], indicating that RBGO1 ADC is likely to be well tolerated. This is of particular significance given that RAGE is also expressed in the adult lung, which might lead to concern regarding the safe use of a RAGE-ADC. However, Gefter and colleagues have recently illustrated that lung isoforms possess distinct epitopes which are not found elsewhere. They suggest that those RAGE isoforms unique to the lung may exhibit both structural and functional differences [16]. However, it is currently unclear as to the specific mechanisms which give rise to any lung-restricted isoforms. When the toxicity of RBGO1-ADC was tested in vivo, no on-target toxicities were observed in the lung of the treated animals. This suggests that either RBGO1-ADC may not target membrane bound RAGE isoforms expressed in pulmonary tissues or that sRAGE expressed by alveolar type I epithelial cells acts as a decoy kidnapping the drug and hence exerting a protective effect. Therefore, it is possible that these soluble variants of RAGE block the RAGE-ADCs avoiding damage to healthy tissues.

Finally, evaluation of the efficacy of RBGO1-ADC within a murine xenograft model demonstrated a significant reduction in tumour growth rate compared to control animals. Whilst a similar reduction in growth rate was also observed within animals treated with mcF alone, the advantage of using a targeted therapeutic approach to avoid systemic toxicity associated with the use of chemotherapeutics such as the auristatins is well documented.

## Conclusions

In summary, we show that RAGE is a suitable target for the development of anti-cancer therapeutics. Additionally, its differential expression between EC and non-malignant cells would make it a suitable target for the development of companion diagnostics. Our data imply the suitability of an ADC approach as they also highlight the importance of native protein binding affinity when designing antibodies for this purpose and suggest a role for receptor activation in effective ADC design. In particular, we demonstrate the efficacy of our RAGE targeting ADC based on the V-region binding, RBGO1 ADC, which was up to 200-fold more efficacious than treatment with cytotoxic drug alone. Initial toxicity evaluation suggests a likely low toxicity and local delivery of RAGE-ADCs to the endometrium could render this targeted therapy safe enough to be quickly directed towards the clinic. In addition, through the murine xenograft model, we demonstrate that RBGO1 ADC effectively reduces tumour growth and is therefore a suitable candidate for further pre-clinical and potential clinical development. Future work will continue the pre-clinical development of ADCs based on the RBGO1 antibody, together with efforts to elucidate important design characteristics of ADCs that might have applicability for multiple targets. Such findings could be translated to novel therapeutics for endometrial cancer patients, providing rational strategies for targeting chemotherapeutic drugs to cells expressing RAGE receptors.

## Supplementary information

**Additional file 1.** Supplemental methods.

**Additional file 2:** **Table S1.** Patient Demographics.

**Additional file 3:** **Figure S1.** Increased RAGE expression in Type II EC is correlated with poorer survival. Endometrial biopsies obtained from patients with a confirmed diagnosis of endometrial cancer (Type II, *n* = 37) were formalin-fixed and paraffin-embedded.

**Additional file 4:** **Figure S2.** RAGE is over expressed in EC cell lines.

**Additional file 5:** **Figure S3.** RAGE expression is absent or very low in healthy human tissues with the exception of lung tissue.

**Additional file 6:** **Figure S4.** Conjugation of auristatins to antibodies used cleavable or non-cleavable linkers.

**Additional file 7:** **Figure S5.** Conjugation of antimitotic agents to each of the 4 anti-RAGE antibodies resulted in a significant level of drug loading.

**Additional file 8:** **Figure S6.** RBGO2, RBGO3 and RBGO4 preferentially target endometrial cancer cells and increase drug sensitivity by up to 40-fold.

**Additional file 9:** **Figure S7.** Blocking experiments confirm the specificity of RBGO1 for RAGE.

**Additional file 10:** **Table S2.** Animal full blood counts.

**Additional file 11:** **Table S3.** Animal histopathology report.

## Data Availability

All data generated or analysed during this study are included in this published article and its supplementary information files.

## Abbreviations

ABMUHB: Abertawe Bro Morgannwg University Health Board
ADC: Antibody-drug conjugate
ANOVA: Analysis of variance
AST: Aspartate aminotransferase
BMI: Body Mass Index
BSA: Bovine serum albumin
DAR: Drug to antibody ratio
DMEM/F12: Dulbecco’s modified eagle medium/Ham’s nutrient mix F-12
DPBS: Dulbecco’s phosphate buffered saline
EC: Endometrial cancer
ECCAC: European Collecion of Authenticated Cell Cultures
ELISA: Elzyme Linked Immunosorbant Assay
ER: Estrogen receptor
ESC: endometrial stromal cells
FBS: Foetal bovine serum
FFPE: Formalin fixed parafin embedded
FITC: Fluorescene isothiocyante
HER: Human epidermal growth factor receptor
KLH: Keyhole limpet haemocyanin
LD_50_: 50% Lethal dose
MC: Maleimido caproyl
MMAE: Monomethyl auristatin E
MMAF: Monomethyl auristatin F
NF-ƙB: Nuclear factor kappa B
PCC: Pearson’s correlation coefficient
PMB: Post menopausal bleeding
PR: Progesterone receptor
qPCR: Quantitative polymerase chain reaction
RAGE: Receptor for advanced glycation end products
SD: Standard deviation
VC: Valine-citrulline
