# Supplementary material for: Antibody drug conjugates against the receptor for advanced glycation end products (RAGE), a novel therapeutic target in endometrial cancer
Source: J Immunother Cancer. 2019 Oct 29;7:280. doi: 10.1186/s40425-019-0765-z (PMC6820928; doi:10.1186/s40425-019-0765-z)
Supplement: Supplementary file 1 — Additional file 1. Supplemental methods. [file 40425_2019_765_MOESM1_ESM.docx]

**Supplemental methods**

*Cell culture*

Primary endometrial cells were isolated as previously described (1). Purified endometrial cells were grown to 80% confluence before passage in complete medium, which comprised a 1:1 mixture of Dulbecco’s Modified Eagle Medium and Ham’s F-12 nutrient medium (DMEM/F12, Thermo Fisher, Gloucester, UK) supplemented with 10 % heat inactivated foetal bovine serum (FBS, Thermo Fisher), 100 units/ml penicillin and 100 µg/ml streptomycin (Thermo Fisher). Cells were maintained in a humidified, 5 % CO_2_ in air atmosphere incubator at 37 °C, and culture medium changed every 48 h.

Cell lines: HEC1A, HEC1B, HEC50 and Ishikawa, were obtained from The European Collection of Authenticated Cell Cultures (ECACC, Public Health England, UK). Cells were grown to 80% confluence before passage in complete medium, maintained in a humidified, 5 % CO_2_ in air atmosphere incubator at 37 °C, and culture medium changed every 48 h.

*Antibody-drug conjugation*

Murine antibodies against RAGE or HER2 were reconstituted in 10 mM Tris/Cl (Sigma), 2 mM EDTA (Sigma) pH 8.0. Antibodies were reduced with 3.5 equivalents TCEP:Ab (10 mM in water, Sigma) for 2 h at 37°C. Without purification the reduced antibody was split in two equal volume aliquots and each aliquot alkylated with 6.5 equivalents of drug linker:Ab (10 mM MMAE or MMAF in DMA with additional DMA added to achieve 5 % v/v final DMA, ADC Biotechnology, St Asaf, UK) for 2 h at 22°C. Following alkylation, N-acetyl cysteine (Sigma) was used to quench any unreacted toxin linker. The conjugates were purified using a HiTrap G25 column (GE Lifesciences) equilibrated in 5 mM histidine/Cl, 50 mM trehalose (Sigma), 0.01% w/v polysorbate 20 (Sigma), pH 6.0. Conjugates were analysed by size exclusion chromatography (SEC) for monomeric content and concentration using a calibration curve of naked antibody. Running conditions: Agilent 1100 HPLC, TOSOH TSK gel G3000SWXL 7.8 mm x 30 cm, 5 μm column (Tosoh Bioscience), 0.5 ml/min in, 0.2 M Potassium Phosphate, 0.25 M Potassium Chloride, 10% IPA, pH 6.95. Drug loading of the conjugates was confirmed using a combination of hydrophobic interaction chromatography (HIC) and reverse phase chromatography. HIC was carried out using a TOSOH Butyl-NPR 4.6 mm x 3.5 cm, 2.5 μm column (Tosoh Bioscience) run at 0.8 ml/min with a 12 min linear gradient between A - 1.5 M (NH4)2SO4, 25 mM NaPi, pH 6.95±0.05 and B - 75% 25 mM NaPi, pH 6.95±0.05, 25% IPA. Reverse phase analysis was performed on a Polymer Lab’s polymeric reversed phase (PLRP) 2.1 mm x 5 cm, 5 μm column (Tosoh Bioscience)run at 1 ml/min at 80°C with a 25 min linear gradient between 0.05% TFA/H_2_O and 0.04% TFA/CH_3_CN. Samples were first reduced by incubation with 1, 4-Dithiothritol (DTT, Sigma) at pH 8.0 at 37°C for 15 min.

*Epitope mapping*

Epitope mapping was performed by PEPperPRINT GmbH, Heidelberg, Germany. The RAGE protein sequence corresponding to the *Homo sapiens* ‘advanced glycosylation end product-specific isoform 1,’ was elongated with neutral GSGSGSG linkers at the C- and N-terminus to avoid the production of truncated peptides. The elongated sequence was then translated into 15 aa peptides with a peptide-peptide overlap of 14 aa. The resulting peptide microarray contained 404 different peptides, each printed in duplicate (808 spots) and framed by additional HA (YPYDVPDYAG) and Flag (DYKDDDDKAS) control peptides (82 spots for each control). Six arrays were produced to enable incubation with the secondary antibody only, so that background interactions could be investigated, and replicates of the main assay to be performed (n = 5). Briefly, arrays were blocked with MB-070 (Rockland Immunochemicals Inc, PA, USA) diluted 1:1 in PBS, pH 7.4 with 0.05% Tween 20 (blocking buffer) for 30 min. Arrays were washed with PBS, pH 7.4 with 0.05% Tween 20 (wash buffer) for 3 x 1 min and then 2 x 10 s before each assay. RBGO1 antibody at concentrations of 1, 10 and 100 µg/ml were incubated on the microarray in wash buffer containing 10% blocking buffer (incubation buffer) for 16 h at 4°C and shaking at 140 rpm. Peptide arrays were washed and then incubated with secondary antibody (goat anti-mouse IgG, H+L, DyLight680), which was added in incubation buffer at a dilution of 1:5000 for 45 min at RT. After imaging of the peptide arrays, control antibody (mouse anti-HA-DyLight800) was added in incubation buffer at a dilution of 1:2000 for 45 min at RT to visualise the control peptides. Images were acquired on a LI-COR Odyssey Imaging System with a scanning offset of 0.65 mm, resolution of 21 µm and scanning intensities of 7/7 (red/green).

Quantification of spot intensities and peptide annotation was done using 16-bit gray scale tiff files at scanning intensities of 7 that exhibit a higher dynamic range than 24-bit colour .tif files. Microarray image analysis was done with PepSlide^®^ Analyzer and an intensity map generated based on median intensities for each of the spots.

*Surface plasmon resonance*

Surface plasmon resonance (SPR) reagents used were Series S Sensor Chips (CM5), HBS-EP+ buffer (10 mM Hepes, 150 mM NaCl, 3 mM ethylenediaminetetraacetic acid, and 0.05% Surfactant P20, pH 7.4), amine coupling kits, mouse antibody capture kits and 10 mM glycine-HCl pH 1.7 (all from GE Healthcare, Uppsala, Sweden).

SPR analysis was performed using a Biacore™ T200 system (GE Healthcare) and HBS-EP+ buffer was used as sample and analysis buffer. The analysis temperature and sample compartment were set to 25°C. Immobilisation of α-mouse antibody was performed using the amine coupling kit in accordance with the manufacturer’s instructions. Anti-mouse antibody was immobilised in all flow cells, but flow cells 1 and 3 were used as reference cells for antibodies captured in flow cells 2 and 4. Protein was injected in order of increasing concentration over reference and active flow cells, applying a single cycle kinetics procedure. Following each binding cycle, the surface was regenerated with an injection of regeneration solution, removing any bound antibody. Blank cycles (antibody + buffer injections + regeneration) were performed between each antibody. Data were double referenced by first subtractuib if reference flow cell and then subtraction of blank cycles. Data were fitted to a one to one binding model using Biacore™ T200 evaluation software 2.0.

*Gene expression analysis*

Gene expression analysis was performed according to the MIQE guidelines (2). Total RNA was isolated from cells following lysis in RLT buffer using the RNeasy Mini kit (Qiagen, Manchester, UK), according to the manufacturer’s instructions. Reverse transcription of 1 μg mRNA was performed in a 20 μl reaction volume using the High Capacity cDNA reverse transcription kit (Thermo Fisher), according to the manufacturer’s instructions. Quantitative PCR primers were designed using the Primer-BLAST primer design software (<http://www.ncbi.nlm.nih.gov/tools/primer-blast/>) and validated by BLAST analysis against the *Homo sapiens* (taxid:9606) Refseq mRNA database. The *RAGE* primers and *GAPDH* and *RPL19* reference gene primers were obtained from Sigma–Aldrich and were as follows: *RAGE* forward, 5’-CAGTGTGGCTCGTGTCCTTC-3’, reverse, 5’-GTCTCCTTTCCATTCCTGTTCATTG-3’; *GAPDH* forward, 5’-GTCCACTGGCGTCTTCAC-3’, reverse, 5’-CTTGAGGCTGTTGTCATACTT-3’; *RPL19* forward, 5’-CCTGTACGGTCCATTC-3’, reverse, 5’-AATCCTCATTCTCCTCATCC-3’. Quantitative PCR was performed in a 10 μl reaction volume comprising 1 × iTaq Universal SYBR Green Supermix (Bio-Rad) with primers added in nuclease-free water to a final concentration of 0.4 mM and 2 μl of cDNA. Thermal cycling parameters were as follows: one cycle of 95 °C for 5 min, followed by 40 cycles of 95 °C for 10 s and 60 °C for 60 s. The expression of each gene was normalised against the geometric mean of the reference genes *GAPDH* and *RPL19*, which were invariant across treatment groups (3), and the relative quantification method was employed to quantify target gene mRNA within samples (4). To generate standard curves, total RNA extracted from cells was reverse transcribed to cDNA, as described. Ten-fold serial dilutions of this reference cDNA were prepared (neat to 1 × 10^-3^) in nuclease-free water (Qiagen). For each sample, target and reference gene mRNA abundance was determined from the appropriate standard curve (quantification cycle, Cq). Changes in mRNA abundance between samples were then determined from the ratio of the target gene Cq to the reference gene Cq.

*Protein expression analysis*

Proteins were normalized to 1 µg/µl using the DC Assay (Bio-Rad) and separated (10 µg per lane) using 10% (v/v) SDS-PAGE. Pre-stained molecular weight markers (Bio-Rad) were run in parallel lanes. After electrophoresis, proteins were transferred to a polyvinylidene difluoride membrane (Bio-Rad); nonspecific sites were blocked using a solution of 5 % (wt/v) bovine serum albumin (Sigma-Aldrich) in Tris-buffered saline and 0.1 % Tween 20 (TBS/T, pH 7.6) overnight at 4 °C with gentle agitation. Membranes were probed with α-RAGE antibody (Santa Cruz Biotechnology, TX, USA, H-300). The antibody used was selected based on recognition of immunoreactive proteins of appropriate molecular weight. Primary antibodies were used at 1:500 dilutions in 5 % (wt/vol) BSA in TBS/T for 2 h with gentle agitation. After incubation, membranes were washed three times for 5 min in TBS/Tween. Membranes were then incubated in secondary horseradish peroxidase-conjugated antibody (Cell Signalling Technology, Danvers, MA) in 5 % (wt/vol) BSA in TBS/T for 2 h and washed three times for 5 min in TBS/T. Steady-state levels of immunoreactive proteins were visualized using enhanced chemiluminescence (Western C; Bio-Rad). Protein loading was evaluated and normalised by examining GAPDH protein levels using a GAPDH antibody (Santa Cruz, FL-335).

**References**

1. Margarit L, Gonzalez D, Lewis PD, Hopkins L, Davies C, Conlan RS, et al. L-selectin ligands in human endometrium: comparison of fertile and infertile subjects. Hum Reprod [Internet]. 2009/07/25. 2009;24:2767–77. Available from: http://www.ncbi.nlm.nih.gov/pubmed/19625313

2. Bustin SA, Benes V, Garson JA, Hellemans J, Huggett J, Kubista M, et al. The MIQE Guidelines: Minimum Information for Publication of Quantitative Real-Time PCR Experiments. Clin Chem [Internet]. 2009;55:611–22. Available from: http://www.clinchem.org/content/55/4/611.abstract

3. Vandesompele J, De Preter K, Pattyn F, Poppe B, Van Roy N, De Paepe A, et al. Accurate normalization of real-time quantitative RT-PCR data by geometric averaging of multiple internal control genes. Genome Biol. 2002/08/20. 2002;3:RESEARCH0034.

4. Nolan T, Hands RE, Bustin SA. Quantification of mRNA using real-time RT-PCR. Nat Protoc. 2007/04/05. 2006;1:1559–82.
