# Supplementary material for: Antibody drug conjugates against the receptor for advanced glycation end products (RAGE), a novel therapeutic target in endometrial cancer
Source: J Immunother Cancer. 2019 Oct 29;7:280. doi: 10.1186/s40425-019-0765-z (PMC6820928; doi:10.1186/s40425-019-0765-z)
Supplement: Supplementary file 2 — Additional file 2: Table S1. Patient Demographics. [file 40425_2019_765_MOESM2_ESM.docx]

**Supplemental Table 1**

| **Age** | **BMI** | **Parity** | **Smoker** | **Diabetic** | **Pathology** | **Cancer type** | **Stage** | **Grade** | **LVSI** | **Chemotherapy** | **Radiotherapy** | **Overall Survival (months)** | **Disease Free period (months)** | **RAGE expression**  **(H-score)** |
| --- | --- | --- | --- | --- | --- | --- | --- | --- | --- | --- | --- | --- | --- | --- |
| 71 | 27 | Y | N | N | Prolapse | - | - | - | - | - | - | 60 | - | 0.2 |
| 47 | 21 | Y | Y | Y | Hysterectomy | - | - | - | - | - | - | 60 | - | 1 |
| 64 | 25 | N | N | N | Prolapse | - | - | - | - | - | - | 60 | - | 0.2 |
| 54 | 22 | Y | Y | N | Post menopausal bleeding | - | - | - | - | - | - | 60 | - | 0.2 |
| 66 | 35 | Y | N | N | Prolapse | - | - | - | - | - | - | 60 | - | 0.2 |
| 54 | 32 | Y | N | N | Prolapse | - | - | - | - | - | - | 60 | - | 0.2 |
| 50 | 30 | Y | N | N | Post menopausal bleeding | - | - | - | - | - | - | 60 | - | 0.2 |
| 62 | 25 | N | N | N | Post menopausal bleeding | - | - | - | - | - | - | 60 | - | 0.2 |
| 49 | 30 | N | N | Y | Hysterectomy | - | - | - | - | - | - | 60 | - | 1 |
| 49 | 18 | Y | N | N | Post menopausal bleeding | - | - | - | - | - | - | 60 | - | 0.2 |
| 73 | 26 | Y | Y | N | Post menopausal bleeding | - | - | - | - | - | - | 60 | - | 0.2 |
| 51 | 39 | Y | N | N | Hysterectomy | - | - | - | - | - | - | 60 | - | 0.4 |
| 50 | 23 | Y | N | N | Hysterectomy | - | - | - | - | - | - | 60 | - | 0.2 |
| 56 | 40 | Y | N | N | Prolapse | - | - | - | - | - | - | 60 | - | 0.4 |
| 56 | 20 | N | N | N | Post menopausal bleeding | - | - | - | - | - | - | 60 | - | 0.2 |
| 51 | 24 | Y | Y | Y | Post menopausal bleeding | - | - | - | - | - | - | 60 | - | 0.2 |
| 54 | 30 | Y | N | Y | Post menopausal bleeding | - | - | - | - | - | - | 60 | - | 0.2 |
| 64 | 38 | Y | N | N | Prolapse | - | - | - | - | - | - | 60 | - | 0.2 |
| 59 | 42 | Y | N | Y | Prolapse | - | - | - | - | - | - | 60 | - | 0.8 |
| 56 | 36 | Y | Y | N | Post menopausal bleeding | - | - | - | - | - | - | 60 | - | 0.4 |
| 63 | 21 | Y | N | Y | Post menopausal bleeding | - | - | - | - | - | - | 60 | - | 0.2 |
| 56 | 32 | Y | N | N | Prolapse | - | - | - | - | - | - | 60 | - | 0.2 |
| 63 | 38 | Y | Y | N | Prolapse | - | - | - | - | - | - | 60 | - | 0.4 |
| 53 | 25 | Y | N | N | Post menopausal bleeding | - | - | - | - | - | - | 60 | - | 0.2 |
| 84 | 24 | N | N | N | Post menopausal bleeding | - | - | - | - | - | - | 60 | - | 0.2 |
| 48 | 31 | Y | N | N | Postmenopausal normal | - | - | - | - | - | - | 60 | - | 0.4 |
| 64 | 27 | Y | N | N | Postmenopausal normal | - | - | - | - | - | - | 60 | - | 0.4 |
| 51 | 30 | Y | N | N | Postmenopausal normal | - | - | - | - | - | - | 60 | - | 0.4 |
| 52 | 25 | Y | N | N | Postmenopausal normal | - | - | - | - | - | - | 60 | - | 0.2 |
| 49 | 18 | Y | N | Y | Postmenopausal normal | - | - | - | - | - | - | 60 | - | 1 |
| 73 | 26 | Y | Y | N | Postmenopausal normal | - | - | - | - | - | - | 60 | - | 0.5 |
| 86 | 34 | Y | N | N | Postmenopausal normal | - | - | - | - | - | - | 60 | - | 0.8 |
| 51 | 39 | Y | N | N | Postmenopausal normal | - | - | - | - | - | - | 60 | - | 0.6 |
| 50 | 23 | Y | N | N | Postmenopausal normal | - | - | - | - | - | - | 60 | - | 0.2 |
| 56 | 40 | Y | N | N | Postmenopausal normal | - | - | - | - | - | - | 60 | - | 0.6 |
| 56 | 20 | N | N | N | Postmenopausal normal | - | - | - | - | - | - | 60 | - | 0.8 |
| 51 | 45 | Y | Y | N | Postmenopausal normal | - | - | - | - | - | - | 60 | - | 0.2 |
| 54 | 30 | Y | N | N | Postmenopausal normal | - | - | - | - | - | - | 60 | - | 0.4 |
| 64 | 38 | Y | N | N | Postmenopausal normal | - | - | - | - | - | - | 60 | - | 0.4 |
| 59 | 40 | Y | N | N | Postmenopausal normal | - | - | - | - | - | - | 60 | - | 0.8 |
| 56 | 36 | Y | Y | N | Postmenopausal normal | - | - | - | - | - | - | 60 | - | 0.4 |
| 63 | 21 | Y | N | N | Postmenopausal normal | - | - | - | - | - | - | 60 | - | 0.2 |
| 56 | 32 | Y | N | N | Postmenopausal normal | - | - | - | - | - | - | 60 | - | 0.4 |
| 65 | 42 | Y | N | N | Postmenopausal normal | - | - | - | - | - | - | 60 | - | 0.6 |
| 63 | 38 | Y | Y | N | Postmenopausal normal | - | - | - | - | - | - | 60 | - | 0.4 |
| 50 | 31 | Y | N | N | Postmenopausal normal | - | - | - | - | - | - | 60 | - | 0.4 |
| 52 | 22 | N | N | N | Postmenopausal normal | - | - | - | - | - | - | 60 | - | 0.2 |
| 55 | 38 | Y | N | N | Postmenopausal normal | - | - | - | - | - | - | 60 | - | 0.5 |
| 73 | 31 | Y | N | N | Postmenopausal normal | - | - | - | - | - | - | 60 | - | 0.5 |
| 49 | 31 | Y | Y | N | Postmenopausal normal | - | - | - | - | - | - | 60 | - | 0.4 |
| 49 | 30 | Y | N | N | Postmenopausal normal | - | - | - | - | - | - | 60 | - | 0.4 |
| 70 | 27 | Y | N | N | Postmenopausal normal | - | - | - | - | - | - | 60 | - | 0.4 |
| 66 | 36 | Y | N | N | Postmenopausal normal | - | - | - | - | - | - | 60 | - | 0.4 |
| 54 | 41 | Y | Y | N | Postmenopausal normal | - | - | - | - | - | - | 60 | - | 0.6 |
| 49 | 33 | Y | Y | Y | Postmenopausal normal | - | - | - | - | - | - | 60 | - | 1 |
| 55 | 27 | N | N | Y | Postmenopausal normal | - | - | - | - | - | - | 60 | - | 1 |
| 76 | 29 | Y | N | N | Postmenopausal normal | - | - | - | - | - | - | 60 | - | 0.8 |
| 71 | 41 | Y | N | N | Postmenopausal normal | - | - | - | - | - | - | 60 | - | 0.5 |
| 62 | 29 | Y | N | N | Postmenopausal normal | - | - | - | - | - | - | 60 | - | 0.4 |
| 59 | 33 | Y | Y | N | Postmenopausal normal | - | - | - | - | - | - | 60 | - | 0.4 |
| 81 | 37 | N | N | N | Postmenopausal normal | - | - | - | - | - | - | 60 | - | 0.8 |
| 79 | 31 | N | N | N | Postmenopausal normal | - | - | - | - | - | - | 60 | - | 0.8 |
| 74 | 25 | Y | N | N | Postmenopausal normal | - | - | - | - | - | - | 60 | - | 0.4 |
| 81 | 24 | Y | N | N | Postmenopausal normal | - | - | - | - | - | - | 60 | - | 0.8 |
| 83 | 26 | Y | Y | N | Postmenopausal normal | - | - | - | - | - | - | 60 | - | 0.8 |
| 61 | 48 | Y | N | N | Postmenopausal normal | - | - | - | - | - | - | 60 | - | 0.8 |
| 64 | 35 | Y | N | N | Postmenopausal normal | - | - | - | - | - | - | 60 | - | 0.4 |
| 59 | 33 | Y | N | N | Postmenopausal normal | - | - | - | - | - | - | 60 | - | 0.4 |
| 66 | 37 | Y | Y | N | Postmenopausal normal | - | - | - | - | - | - | 60 | - | 0.4 |
| 81 | 42 | Y | N | N | Postmenopausal normal | - | - | - | - | - | - | 60 | - | 1 |
| 60 | 35 | Y | N | Y | Endometrial cancer | 1 | 1A | 1 | N | N | N | 60 | 60 | 1 |
| 41 | 31 | Y | N | N | Endometrial cancer | 1 | 1A | 2 | N | N | N | 60 | 60 | 0.2 |
| 72 | 57 | Y | N | Y | Endometrial cancer | 1 | 1A | 1 | N | N | N | 60 | 60 | 5 |
| 76 | 28 | Y | N | Y | Endometrial cancer | 1 | 1B | 2 | Y | N | Brachytherapy | 60 | 60 | 3 |
| 83 | 25 | N | N | Y | Endometrial cancer | 1 | 1B | 2 | N | N | Offered | 12 | 12^*^ | 5 |
| 64 | 32 | N | N | Y | Endometrial cancer | 1 | 1A | 1 | N | N | N | 60 | 60 | 0.2 |
| 38 | 42 | N | N | N | Endometrial cancer | 1 | 1B | 1 | N | N | Brachytherapy | 60 | 60 | 0.2 |
| 85 | 29 | N | N | N | Endometrial cancer | 1 | 1B | 2 | N | N | Brachytherapy | 60 | 60 | 1.5 |
| 66 | 42 | Y | N | N | Endometrial cancer | 1 | 1A | 2 | Y | N | Brachytherapy | 60 | 60 | 2 |
| 68 | 44 | N | Y | N | Endometrial cancer | 1 | 1B | 2 | N | N | Brachytherapy | 60 | 60 | 2.5 |
| 73 | 34 | Y | N | N | Endometrial cancer | 1 | 1B | 2 | N | N | Brachytherapy | 60 | 60 | 2 |
| 81 | 27 | Y | Y | N | Endometrial cancer | 1 | 1B | 2 | N | N | Brachytherapy | 12 | 12^*^ | 5 |
| 62 | 33 | N | N | Y | Endometrial cancer | 1 | 1A | 1 | N | N | N | 60 | 60 | 0.2 |
| 44 | 29 | Y | N | N | Endometrial cancer | 1 | 1A | 2 | N | N | N | 60 | 60 | 0.2 |
| 76 | 48 | N | N | Y | Endometrial cancer | 1 | 1A | 2 | N | N | N | 60 | 60 | 4 |
| 76 | 26 | Y | N | N | Endometrial cancer | 1 | 1B | 1 | Y | N | Brachytherapy | 60 | 60 | 1 |
| 82 | 22 | Y | N | Y | Endometrial cancer | 1 | 1B | 2 | N | N | Brachytherapy | 12 | 12^*^ | 4 |
| 65 | 20 | Y | Y | Y | Endometrial cancer | 1 | 1A | 1 | N | N | N | 60 | 60 | 0.2 |
| 39 | 44 | N | N | N | Endometrial cancer | 1 | 1B | 1 | N | N | Brachytherapy | 60 | 60 | 2 |
| 84 | 31 | N | N | N | Endometrial cancer | 1 | 1B | 1 | N | N | Brachytherapy | 60 | 60 | 1 |
| 67 | 42 | N | N | Y | Endometrial cancer | 1 | 1A | 2 | Y | N | Brachytherapy | 60 | 60 | 3 |
| 68 | 47 | N | N | Y | Endometrial cancer | 1 | 1B | 1 | N | N | Brachytherapy | 60 | 60 | 3 |
| 74 | 35 | Y | N | N | Endometrial cancer | 1 | 1B | 2 | N | N | Brachytherapy | 60 | 60 | 2 |
| 82 | 28 | Y | N | Y | Endometrial cancer | 1 | 1B | 1 | N | N | Brachytherapy | 12 | 12^*^ | 3 |
| 77 | 32 | Y | N | N | Endometrial cancer | 1 | 1B | 2 | Y | N | EBRT and brachytherapy | 36 | 36 | 4 |
| 64 | 20 | N | N | N | Endometrial cancer | 1 | 1B | 2 | N | N | Brachytherapy | 36 | 36 | 2.5 |
| 74 | 25 | N | Y | N | Endometrial cancer | 1 | 1B | 2 | Y | N | EBRT and brachytherapy | 36 | 36 | 4 |
| 81 | 27 | Y | N | N | Endometrial cancer | 1 | 1B | 2 | N | N | Brachytherapy | 36 | 36 | 3 |
| 60 | 24 | Y | N | N | Endometrial cancer | 1 | 2 | 1 | Y | N | EBRT and brachytherapy | 48 | 48 | 2 |
| 76 | 24 | Y | N | N | Endometrial cancer | 1 | 1b | 2 | Y | N | Brachytherapy | 36 | 36 | 4 |
| 59 | 50 | Y | N | Y | Endometrial cancer | 1 | 1a | 1 | N | N | N | 24 | 24 | 4 |
| 77 | 22 | Y | N | N | Endometrial cancer | 1 | 1B | 1 | N | N | Brachytherapy | 24 | 24 | 2 |
| 60 | 48 | N | N | N | Endometrial cancer | 1 | 1A | 1 | N | N | N | 24 | 24 | 2.5 |
| 55 | 40 | Y | N | N | Endometrial cancer | 1 | 1B | 1 | N | N | Brachytherapy | 24 | 24 | 2 |
| 89 | 31 | N | N | N | Endometrial cancer | 1 | 1A | 1 | N | N | N | 24 | 24 | 2 |
| 50 | 45 | Y | N | N | Endometrial cancer | 1 | 3c | 2 | Y | Carboplatin and adj cisplatin | ERBT, brachytherapy and aortic nodes | 24 | 24 | 4 |
| 27 | 77 | N | N | N | Endometrial cancer | 1 | 1A | 1 | Y | N | N | 24 | 24 | 4 |
| 76 | 40 | N | N | N | Endometrial cancer | 1 | 2 | 2 | N | N | Brachytherapy | 24 | 24 | 4 |
| 60 | 22 | Y | N | N | Endometrial cancer | 1 | 1A | 2 | N | N | N | 24 | 24 | 3 |
| 37 | 36 | N | N | N | Endometrial cancer | 1 | 2 | 1 | N | N | EBRT and brachytherapy | 24 | 24 | 1.5 |
| 45 | 54 | N | N | N | Endometrial cancer | 1 | 1A | 1 | N | N | N | 60 | 60 | 2 |
| 67 | 36 | Y | N | N | Endometrial cancer | 1 | 1A | 1 | N | N | N | 60 | 60 | 2 |
| 60 | 25 | N | N | N | Endometrial cancer | 1 | 1A | 1 | N | N | N | 60 | 60 | 2 |
| 74 | 28 | N | N | N | Endometrial cancer | 1 | 1A | 1 | N | N | N | 60 | 60 | 2 |
| 58 | 34 | Y | N | Y | Endometrial cancer | 1 | 1A | 1 | N | N |  | 60 | 60 | 3 |
| 58 | 35 | Y | N | N | Endometrial cancer | 1 | 1B | 2 | Y | N | Brachytherapy | 60 | 60 | 4 |
| 49 | 42 | Y | N | Y | Endometrial cancer | 1 | 2 | 2 | Y | N | EBRT and brachytherapy | 60 | 60 | 5 |
| 75 | 51 | N | N | N | Endometrial cancer | 1 | 1B | 2 | Y | N | Brachytherapy | 60 | 60 | 4 |
| 47 | 62 | Y | Y | N | Endometrial cancer | 1 | 1A | 2 | N | N | N | 60 | 60 | 3.5 |
| 81 | 24 | N | Y | N | Endometrial cancer | 1 | 1B | 2 | N | N | Brachytherapy | 60 | 72* | 3 |
| 91 | 47 | Y | N | N | Endometrial cancer | 1 | 1A | 2 | Y | N | N | 12 | 12* | 5 |
| 67 | 24 | Y | N | N | Endometrial cancer | 1 | 1B | 1 | N | N | Brachytherapy | 60 | 60 | 3 |
| 81 | 40 | Y | N | Y | Endometrial cancer | 1 | 1A | 1 | N | N | N | 60 | 60 | 4 |
| 55 | 31 | N | N | N | Endometrial cancer | 1 | 1A | 1 | N | N | N | 60 | 60 | 2 |
| 61 | 31 | Y | Y | N | Endometrial cancer | 2 | 1A | Clear cell 3 | N | Cisplatin + Taxane | EBRT and Brachytherapy | 60 | 60 | 2 |
| 73 | 28 | Y | N | N | Endometrial cancer | 2 | 1A | MMMT | N | Cisplatin + Taxane | N | 60 | 60 | 1 |
| 79 | 49 | Y | Y | Y | Endometrial cancer | 2 | 4B | Serous | Y | Y | N | 14 | 0 | 5 |
| 79 | 50 | Y | N | N | Endometrial cancer | 2 | 4B | Serous | Y | Y | N | 15 | 0 | 5 |
| 78 | 28 | Y | N | N | Endometrial cancer | 2 | 2 | Serous | Y | N | EBRT and Brachytherapy | 25 | 12 | 3 |
| 75 | 26 | Y | N | N | Endometrial cancer | 2 | 1C | Serous | Y | N | EBRT and Brachytherapy | 30 | 29 | 2 |
| 80 | 34 | Y | N | Y | Endometrial cancer | 2 | 2 | Serous | Y | Cisplatin + Taxane | EBRT and Brachytherapy | 22 | 20 | 3 |
| 73 | 27 | Y | N | N | Endometrial cancer | 2 | 1A | MMMT | N | Cisplatin + Taxane | N | 60 | 48 | 1 |
| 61 | 31 | Y | Y | N | Endometrial cancer | 2 | 1A | Clear cell 3 | N | Cisplatin + Taxane | EBRT and Brachytherapy | 60 | 60 | 1 |
| 71 | 31 | N | N | N | Endometrial cancer | 2 | 3B | MMMT | Y | Cisplatin + Taxane | EBRT and Brachytherapy | 17 | 0 | 2 |
| 68 | 24 | N | N | Y | Endometrial cancer | 2 | 4B | Serous | Y | Carboplatin-Taxol | N | 18 | 0 | 4 |
| 66 | 22 | Y | Y | N | Endometrial cancer | 2 | 1B | MMMT | Y | Carboplatin-Taxol | EBRT and Brachytherapy | 23 | 29 | 1 |
| 78 | 29 | Y | N | N | Endometrial cancer | 2 | 2 | Serous | Y | N | EBRT and Brachytherapy | 25 | 12 | 2 |
| 75 | 27 | Y | Y | N | Endometrial cancer | 2 | 1C | Serous | Y | N | EBRT and Brachytherapy | 30 | 29 | 2.5 |
| 80 | 34 | N | N | Y | Endometrial cancer | 2 | 2 | Serous | Y | Cisplatin + Taxane | EBRT and Brachytherapy | 24 | 20 | 2 |
| 71 | 38 | Y | N | N | Endometrial cancer | 2 | 3B | MMMT | Y | Cisplatin + Taxane | EBRT and Brachytherapy | 18 | 0 | 3 |
| 68 | 26 | N | Y | N | Endometrial cancer | 2 | 4B | Serous | Y | Carboplatin-Taxol | N | 30 | 0 | 4 |
| 66 | 25 | Y | Y | N | Endometrial cancer | 2 | 1B | MMMT | Y | Platinum | EBRT and Brachytherapy | 23 | 29 | 3 |
| 79 | 25 | N | N | N | Endometrial cancer | 2 | 3A | clear cell 3 | Y | Carboplatin | EBRT and Brachytherapy | 18 | 18 | 4 |
| 63 | 30 | Y | N | N | Endometrial cancer | 2 | 1a | SEROUS | N | N | Brachy | 42 | 42 | 3 |
| 75 | 30 | Y | N | N | Endometrial cancer | 2 | 4b | SEROUS | Y | N | N | 16 | 16 | 5 |
| 80 | 32 | Y | N | N | Endometrial cancer | 2 | 1B | Carcinosarcoma | Y | Cisplatin + Taxane | EBRT and Brachytherapy | 35 | 35 | 2 |
| 71 | 28 | Y | N | N | Endometrial cancer | 2 | 4B | Carcinosarcoma | Y | Cisplatin + Taxane | N | 12 | 12 | 5 |
| 76 | 41 | Y | N | Y | Endometrial cancer | 2 | 1B | SEROUS | Y | Carboplatin | EBRT and Brachytherapy | 34 | 34 | 4 |
| 70 | 30 | Y | N | N | Endometrial cancer | 2 | 1B | clear cell 3 | Y | Carboplatin-Taxol | EBRT and Brachytherapy | 34 | 34 | 3 |
| 81 | 27 | Y | N | N | Endometrial cancer | 2 | 1A | MMMT | N | N | N | 60 | 60 | 2 |
| 67 | 28 | Y | Y | N | Endometrial cancer | 2 | 1C | SEROUS | Y | Carbo tx | EBRT and Brachytherapy | 60 | 60 | 3 |
| 81 | 35 | Y | N | Y | Endometrial cancer | 2 | 2 | SEROUS | Y | N | EBRT and Brachytherapy | 10 | 10 | 5 |
| 66 | 28 | N | Y | N | Endometrial cancer | 2 | 4B | SEROUS | Y | Cisplatin + Taxane | N | 52 | 52 | 5 |
| 81 | 33 | Y | N | N | Endometrial cancer | 2 | 1B | Carcinosarcoma | Y | Cisplatin + Taxane | EBRT and Brachytherapy | 24 | 24 | 3.5 |
| 71 | 41 | Y | Y | Y | Endometrial cancer | 2 | 4B | SEROUS | Y | Cisplatin + Taxane | N | 2 | 2 | 5 |
| 75 | 32 | N | N | N | Endometrial cancer | 2 | 3B | MMMT | Y | Cisplatin + Taxane | EBRT and Brachytherapy | 22 | 22 | 5 |
| 66 | 29 | Y | Y | N | Endometrial cancer | 2 | 1C | SEROUS | Y | Carboplatin-Taxol | EBRT and Brachytherapy | 60 | 60 | 3.5 |
| 71 | 29 | Y | N | N | Endometrial cancer | 2 | 1A | MMMT | N | N | N | 60 | 60 | 2 |
| 66 | 27 | N | Y | N | Endometrial cancer | 2 | 4B | SEROUS | Y | Cisplatin + Taxane | N | 30 | 30 | 5 |
| 68 | 26 | Y | Y | N | Endometrial cancer | 2 | 1B | MMMT | Y | Cisplatin + Taxane | EBRT and Brachytherapy | 60 | 17 | 4 |
| 72 | 34 | Y | N | N | Endometrial cancer | 2 | 1B | Clear cell 3 | Y | Carboplatin-Taxol | EBRT and Brachytherapy | 34 | 34 | 3 |

^*^ Death not due to endometrial cancer
