# Supplementary figures and images for: Antibody drug conjugates against the receptor for advanced glycation end products (RAGE), a novel therapeutic target in endometrial cancer
Source: J Immunother Cancer. 2019 Oct 29;7:280. doi: 10.1186/s40425-019-0765-z (PMC6820928; doi:10.1186/s40425-019-0765-z)

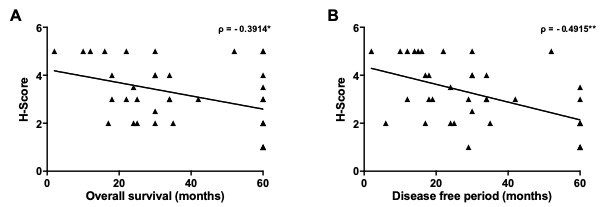

Supplement: Supplementary file 3 — Additional file 3: Figure S1. Increased RAGE expression in Type II EC is correlated with poorer survival. Endometrial biopsies obtained from patients with a confirmed diagnosis of endometrial cancer (Type II, n = 37) were formalin-fixed and paraffin-embedded. [file 40425_2019_765_MOESM3_ESM.jpg]

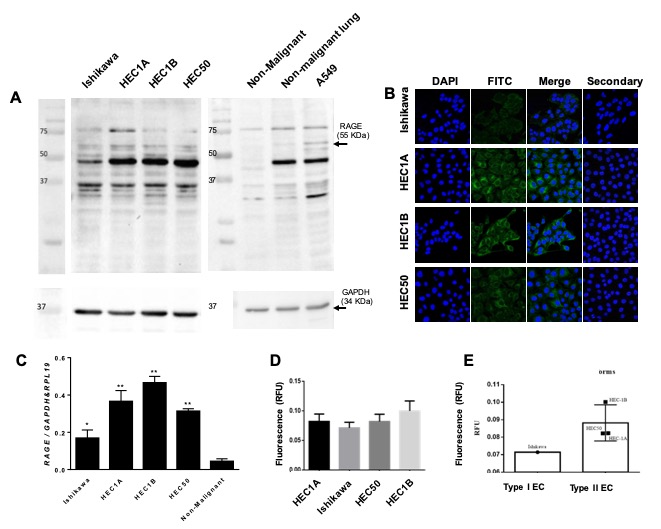

Supplement: Supplementary file 4 — Additional file 4: Figure S2. RAGE is over expressed in EC cell lines. [file 40425_2019_765_MOESM4_ESM.jpg]

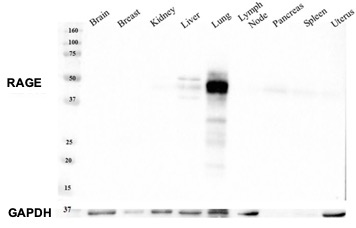

Supplement: Supplementary file 5 — Additional file 5: Figure S3. RAGE expression is absent or very low in healthy human tissues with the exception of lung tissue. [file 40425_2019_765_MOESM5_ESM.jpg]

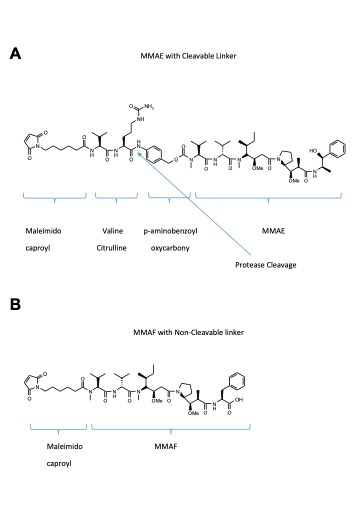

Supplement: Supplementary file 6 — Additional file 6: Figure S4. Conjugation of auristatins to antibodies used cleavable or non-cleavable linkers. [file 40425_2019_765_MOESM6_ESM.jpg]

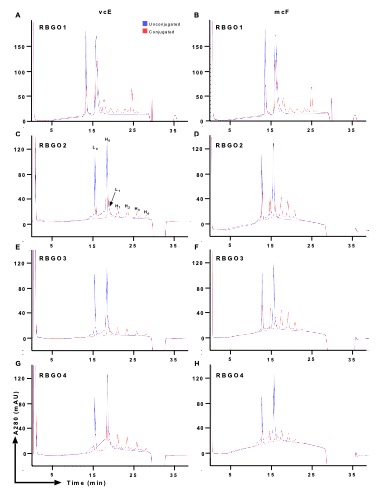

Supplement: Supplementary file 7 — Additional file 7: Figure S5. Conjugation of antimitotic agents to each of the 4 anti-RAGE antibodies resulted in a significant level of drug loading. [file 40425_2019_765_MOESM7_ESM.jpg]

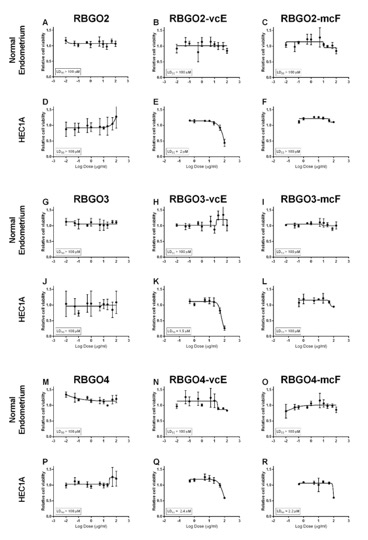

Supplement: Supplementary file 8 — Additional file 8: Figure S6. RBGO2, RBGO3 and RBGO4 preferentially target endometrial cancer cells and increase drug sensitivity by up to 40-fold. [file 40425_2019_765_MOESM8_ESM.jpg]

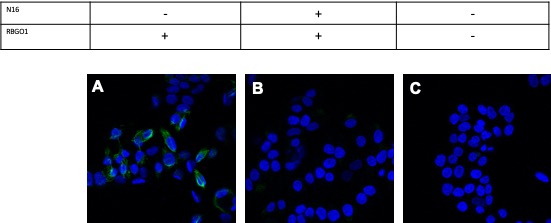

Supplement: Supplementary file 9 — Additional file 9: Figure S7. Blocking experiments confirm the specificity of RBGO1 for RAGE. [file 40425_2019_765_MOESM9_ESM.jpg]
