# Supplementary material for: Antibody drug conjugates against the receptor for advanced glycation end products (RAGE), a novel therapeutic target in endometrial cancer
Source: J Immunother Cancer. 2019 Oct 29;7:280. doi: 10.1186/s40425-019-0765-z (PMC6820928; doi:10.1186/s40425-019-0765-z)
Supplement: Supplementary file 10 — Additional file 10: Table S2. Animal full blood counts. [file 40425_2019_765_MOESM10_ESM.docx]

**Supplemental Table 2**

| **Treatment Group** | **RBC (M/µl)** | **HCT (%)** | **HGB (g/dl)** | **MCV (fl)** | **MCH (pg)** | **MCHC (g/dl)** | **RDW (%)** | **RETIC (%)** | **RETIC (K/µl)** | **WBC (K/µl)** | **NEU (%)** | **LYM (%)** | **MONO (%)** | **EOS (%)** | **BASO (%)** | **PLT (K/µl)** | **MPV (fl)** | **PDW (%)** |
| --- | --- | --- | --- | --- | --- | --- | --- | --- | --- | --- | --- | --- | --- | --- | --- | --- | --- | --- |
| Control | 8.16 ± 0.38 | 41.67 ± 2.43 | 13.42 ± 0.58 | 51.00 ± 1.76 | 16.40 ± 0.36 | 32.17 ± 0.40 | 16.43 ± 0.58 | 5.07 ± 0.67 | 415.95 ± 70.37 | 6.18 ± 2.11 | 69.47 ±2.11 | 27.17 ± 3.01 | 1.77 ± 0.49 | 1.12 ± 0.43 | 0.48 ± 0.28 | 1406.67 ± 30.86 | 10.00 ± 0.43 | 21.83 ± 0.51 |
| RBGO1 ADC (3 mg/kg) | 8.89 ± 0.83 | 44.92 ± 3.25 | 13.83 ± 0.88 | 50.70 ± 1.90 | 15.67 ± 0.76 | 30.90 ± 0.44 | 15.90 ± 0.40 | 4.30 ± 1.11 | 376.58 ± 80.69 | *2.36 ± 0.79 | 63.53 ± 6.65 | 32.43 ± 7.91 | 1.97 ± 0.67 | 0.70 ± 0.35 | 1.40 ± 0.46 | 1486.67 ± 66.44 | 10.02 ± 1.17 | 21.90 ± 2.01 |
| RBGO1 ADC (20 mg/kg) | 8.19 ± 0.85 | 42.25 ± 4.58 | 14.25 ± 1.39 | 51.57 ± 0.61 | 17.47 ± 2.11 | 33.87 ± 3.74 | 16.07 ± 0.46 | *2.95 ± 0.84 | *236.77 ± 45.89 | *1.48 ± 0.03 | 66.00 ± 13.33 | 29.77 ± 13.76 | 1.37 ± 1.12 | 1.43 ± 0.60 | 1.43 ± 1.00 | *1784.17 ± 129.33 | 10.33 ± 0.76 | 21.40 ± 0.90 |

* Significant change from control
