# Supplementary material for: Antibody drug conjugates against the receptor for advanced glycation end products (RAGE), a novel therapeutic target in endometrial cancer
Source: J Immunother Cancer. 2019 Oct 29;7:280. doi: 10.1186/s40425-019-0765-z (PMC6820928; doi:10.1186/s40425-019-0765-z)
Supplement: Supplementary file 11 — Additional file 11: Table S3. Animal histopathology report. [file 40425_2019_765_MOESM11_ESM.docx]

**Supplemental Table 3**

| **Animal number** | **Rx group** | **Bowel** | **Ovary** | **Brain** | **Liver** | **Lungs** | **Uterus** | **Spleen** | **Stomach** | **Bladder** | **Heart** | **Kidney** |
| --- | --- | --- | --- | --- | --- | --- | --- | --- | --- | --- | --- | --- |
| 9 | Control | Normal (includes small bowel and pancreas) | Normal | Normal (cortex and cerebellum) | Single portal tract, mild portal chronic inflammation. | Area of chronic inflammation, including lymphoid follicle formation | Normal | Normal pulp | Normal | Normal | Normal | Focal areas of interstitial chronic inflammation |
| 10 | Control | Normal (includes small bowel and pancreas) | Normal | Normal (includes cerebellum) | Mild lobular inflammation | Normal | Normal | Normal pulp | Normal | Normal | Normal | Normal |
| 11 | Control | Normal (includes small bowel and pancreas) | Normal | Normal (includes cerebellum and hippocampus) | Normal | Normal | Normal | Normal pulp | Normal | Normal | Normal | Renal sinus perivascular chronic inflammation |
|  |  |  |  |  |  |  |  |  |  |  |  |  |
| 13 | SNIPER-ADC (3 mg/kg) | Normal (includes small bowel and pancreas) | Normal | Normal | All three portal tract shows mild chronic inflammation | Peri-bronchial chronic inflammation with lymphoid follicle formation | Normal | Normal pulp | Normal | Normal | Normal (myocardia) | Normal |
| 15 | SNIPER-ADC (3 mg/kg) | Normal (includes small bowel and pancreas) | Normal | Normal | Normal | Normal | Normal | Normal pulp | Normal | Normal | Normal | Normal |
| 19 | SNIPER-ADC (3 mg/kg) | Normal (includes small bowel and pancreas) | Normal | Normal | 1 focus of chronic inflammation around a central vein | Normal | Normal | Normal pulp | Normal | Normal | Normal | Normal |
|  |  |  |  |  |  |  |  |  |  |  |  |  |
| 14 | SNIPER-ADC (20 mg/kg) | Normal (includes small bowel and pancreas) | Normal | Normal (includes cerebellum) | Normal | Normal | Normal | Normal pulp | Normal | Normal | Normal | Renal sinus perivascular chronic inflammation |
| 17 | SNIPER-ADC (20 mg/kg) | Normal (includes small bowel and pancreas) | Normal | Normal | Normal | Peri-bronchial chronic inflammation | Normal | Normal pulp | Normal | Normal | Normal | Normal |
| 20 | SNIPER-ADC (20 mg/kg) | Normal (no pancreas present) | Normal | Normal | 1 Portal tract shows mild portal chronic inflammation | Normal | Normal | Normal pulp | Normal | Normal | Normal | Normal |
